# In Vivo Antibacterial Efficacy of Antimicrobial Peptides Modified Metallic Implants—Systematic Review and Meta-Analysis

**DOI:** 10.1021/acsbiomaterials.1c01307

**Published:** 2022-04-12

**Authors:** Amrit Kaur Sandhu, Ying Yang, Wen-Wu Li

**Affiliations:** School of Pharmacy and Bioengineering, Keele University, Thornburrow Drive, Stoke-on-Trent, ST4 7QB, United Kingdom

**Keywords:** Antimicrobial peptides (AMPs), surface modification, animal, in vivo, metallic implant, biofilm, meta-analysis

## Abstract

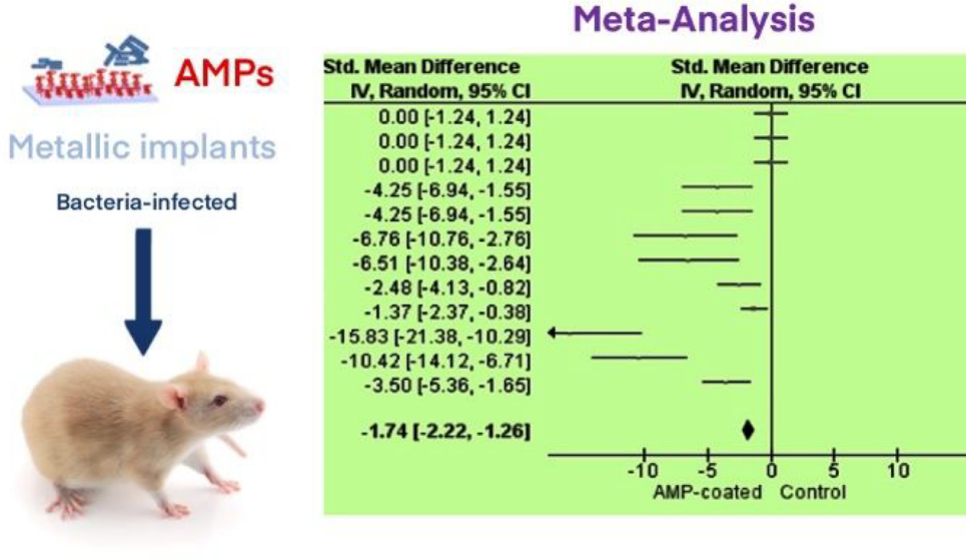

Biomaterial-associated infection is difficult to detect and brings consequences that can lead to morbidity and mortality. Bacteria can adhere to the implant surface, grow, and form biofilms. Antimicrobial peptides (AMPs) can target and kill bacterial cells using a plethora of mechanisms of action such as rupturing the cell membrane by creating pores via depolarization with their cationic and amphipathic nature. AMPs can thus be coated onto metal implants to prevent microbial cell adhesion and growth. The aim of this systematic review was to determine the potential clinical applications of AMP-modified implants through in vivo induced infection models. Following a database search recently up to 22 January 2022 using PubMed, Web of Science and Cochrane databases, and abstract/title screening using the PRISMA framework, 24 studies remained, of which 18 were used in the random effects meta-analysis of standardized mean differences (SMD) to get effect sizes. Quality of studies was assessed using SYRCLE’s risk of bias tool. The data from these 18 studies showed that AMPs carry antibacterial effects, and the meta-analysis confirmed the favorited antibacterial efficacy of AMP-coated groups over controls (SMD −1.74, 95%CI [−2.26, −1.26], *p* < 0.00001). Subgroup analysis showed that the differences in effect size are random, and high heterogeneity values suggested the same. HHC36 and vancomycin were the most common AMPs for surface modification and *Staphylococcus aureus*, the most tested bacterium in vivo. Covalent binding with polymer brush coating and physical layer-by-layer incorporation of AMPs were recognized as key methods of incorporation to achieve desired densities. The use of fusion peptides seemed admirable to incorporate additional benefits such as osteointegration and wound healing and possibly targeting more microbe strains. Further investigation into the incorporation methods, AMP activity against different bacterial strains, and the number of AMPs used for metal implant surface modification is needed to progress toward potential clinical application.

## 1 Introduction

Implant-associated infection poses significant risk to patients where antibiotic treatment is prolonged, follow-up surgeries may be required, and possible amputation may be needed and, in some cases, may lead to morbidity and mortality.^1,2^ Infection can be caused by bacteria adhering to the implant surface or may be due to the lack of immune power at the implant/tissue interface.^3^ Bacteria form biofilms by adhering to the implant surface where these cells continue to add and grow to then form immobile communities.^4^ The immobile communities are able to pass on genetic material that enables antibiotic resistance at high rates while having low metabolic activities and protection from the biofilm matrix making antibiotic treatments difficult.^5^ Moreover, immobile communities mean that routine cultures are unable to detect the infection as the biofilm restricts microbes from leaving the colonies and may further increase chances of bacterial resistance to treatment. Microbial infection centered at the implant surface can cause severe inflammatory reactions which can activate osteoclasts to then result in periprosthetic osteolysis and cause the implant to loosen.^6^ This could mean that the implant is to be removed or replaced, although it is possible that the infection remains in surrounding tissue or even within phagocytes.^7^ As a result, it may be more beneficial to prevent infection in the first place.

Biofilms can form more easily on metal implants such as titanium (Ti), which makes Ti-associated infections more common.^8^ One approach may be to locally deliver antibiotics or antimicrobial peptides (AMPs). AMPs carry the benefit that they are small in size, have a broad spectrum of activity, and are fast acting.^9^ AMPs can be classified as either ribosomally synthesized antimicrobial peptides (rAMPs) or non-ribosomally synthesized antimicrobial peptides (nrAMPs).^10,11^ nrAMPs such as peptide antibiotics, vancomycin (VAN), bacitracin, and polymyxin B are synthesized by peptide synthetase and often found in bacteria and fungi. rAMPs are derived from innate immune responses as effector molecules such as cathelicidin and LL-37.^12^ Analogues of rAMP are also designed and chemically synthesized to improve their antibacterial activities.^13^

There are various mechanisms of action for nrAMP, natural rAMP, and synthetic AMPs.^10,14,15^ nrAMP, such as cationic polymycin and gramicidin S, may share the similar mechanism as cationic rAMP via self-promoted uptake across the cytoplasmic membrane and disruption of the barrier, while VAN inhibits the cell wall synthesis and bacitracin inhibits the transfer of peptidoglycan precursors to bactoprenol pyrophosphate.^10^ For rAMPs and synthetic AMPs, there are three major mechanisms of action: “Barrel-stave”, “Carpet model”, and “Toroidal-pore” where each mechanism generally follows three steps: attraction, attachment, and peptide insertion.^14^ Attraction is where electrostatic bonding takes place, and the peptide is to link with the lipopolysaccharide on bacterial membranes in the attachment step. The peptide should essentially span across the polysaccharide bacterial surface in Gram-negative bacteria. Similarly, with Gram-positive bacteria, peptides should link with teichoic and lipoteichoic acid.^15^ Each mechanism involves an I-state where the peptides are arranged parallel to the lipid membrane surface and in higher concentrations can change their orientation. With the Carpet model, AMPs interact with the acidic lipid-rich regions which are spread out on the membrane to essentially form a carpet to commence cell lysis once peptides begin to form pores in the membrane at the critical threshold concentrations.^16^ AMPs repel the lipid head groups in the Barrel-stave model by facing the hydrophilic end toward them which force them away to thin the membrane.^13^ Upon reaching the threshold concentration, AMPs penetrate further into the pore where the hydrophilic end targets the insides of the pore and the hydrophobic end leans toward the acyl chains.^15^ The toroidal pore model involves AMP molecule adsorption to the bilayer surface which results in the membrane to bend. The newly formed pore is hence a result of the AMPs passing through the bilayer where the AMPs themselves line the pores.^17^ AMPs are also able to help immunomodulatory actions such as encouraging phagocytes to kill bacteria, chemo-attraction of leukocytes, and regulation of immune responses.^18^ AMPs have demonstrated that wound healing, angiogenesis, and osteogenic properties are promising.^19^

Despite the promising antimicrobial effects of AMPs, bacteria can still develop resistance to AMPs through various mechanisms involving both cell wall modifications and change of cellular metabolism.^20^ For nrAMPs such as vancomycin, VAN inhibits the synthesis of cell wall through binding to the d-Ala-d-Ala region of Lipid II, while breakdown of the natural precursor and its replacement with d-Ala-d-Ser or d-Ala-d-lac cause low affinity to VAN and develop resistance.^21^ Although cationic rAMPs are less likely, as they work fast to disrupt membranes, microbes may still become resistant to these AMPs where bacteria can change the charges on their membrane to become more positive or by adding neutral components to the membrane or by changing the membrane fluidity, or via AMP degradation by proteases and efflux pumps.^13,20^ Both nrAMP and rAMPs including synthetic AMPs are generally less prone to resistance comparing antibiotics;^22^ these properties mean that they can be promising antimicrobial agents and immobilized onto metal surfaces by introducing functional groups and nanostructures while still performing.^23^ Bioactive sites, specific to AMPs, can be created such as hydroxyl groups from NaOH treatment, dopamine treatment for a bioactive layer,^24−26^ and NH_2_ groups using (3-aminopropyl) triethoxysilane (APTES).^23,27^

There are only few systematic reviews and meta-analyses of animal studies of antimicrobial coated implants using various inorganic materials and organic compounds rather than the promising AMPs.^28,29^ In this study, a systematic review has been undertaken to find out whether AMP-modified metallic implants showed antibacterial efficacy in in vivo induced infection models and if these AMP-coated implants may have potential in clinical applications. To this end, the different AMPs undergo comparisons, and where relevant, a meta-analysis has been performed to obtain effect size and heterogeneity.

## 2 Methods

### 2.1 Protocol

A Population, Intervention, Comparison, and Outcome (PICO) model^30^ was defined to extract relevant information from each study. Table 1 shows a summary of the data to be extracted and used in relation to the review question. For the inclusion criteria in this model, animal studies (not limited to species) were accepted; studies using implants made from any type of metal treated with AMP used at an induced infection site in vivo were considered for intervention. Comparisons between AMP-coated and uncoated metal implants (control groups) in vivo were looked at and the outcome measures focused on changes in bacterial counts. Exclusion criteria for the PICO model, respectively, were studies not testing in vivo and interventions where AMP-treatment was combined with additional substances (drugs or nanoparticles), and outcome measures were excluded where changes such as inflammatory response were observed instead of microbial changes.

**Table 1:** PICO Model

Population	Animal studies
Intervention	Peptide-treated metal implants at induced infection site
Comparisons	Peptide-coated and uncoated metal implants
Outcome measures	Changes in bacterial counts

### 2.2 Search Strategy

PubMed, Web of Knowledge, and Cochrane databases were all used with their advanced search features initially accessed on 30 Nov 2020 and last updated on 22 January 2022. The keywords used to perform the search were inserted: “(antimicrobial peptide OR antibacterial peptide) AND (implant coating OR surface modification) AND (animal OR in vivo)”. There were no filters used for any database.

### 2.3 Data Collection and Analysis

#### 2.3.1 Selection of Studies

The search results were collated onto RefWorks and then exported onto Microsoft Excel for processing using the Preferred Reporting Items for Systematic reviews and Meta-analysis (PRISMA) framework guidelines to identify relevant papers.^31^ The first step was to remove duplicated articles using the “Primary title” and “Authors” columns to identify these. Using Microsoft Excel, “review” was searched within the file and review papers removed. Titles and abstracts were then screened for relevance using the PICO model identified. Following this, full texts were screened for deeper analysis in relation to the PICO model.

#### 2.3.2 Data Collection and Management

The selection of studies was performed by two individuals, and a third individual was present to advise on any disagreements. A table was put together to compile relevant information from the final studies included.

#### 2.3.3 Assessment of Risk of Bias (ROB) in Included Studies

The Cochrane Collaboration compiles the SYRCLE’s ROB tool^32^ which was used to determine the ROB specific to animal intervention studies. There are ten questions in this ROB tool that are to be considered for each included study where this was used together with signaling questions for appropriate use of the ROB tool. The “yes” response indicates a lower ROB where responses could either be “yes”/”no”/“unclear”.

#### 2.3.4 Measures of Treatment Effect

Studies having three or more animals per group and an untreated control were eligible for the meta-analysis. Results from studies looking at colony forming units (CFU) were used to perform a meta-analysis to compare the effects from different studies. Log CFU counts were converted to CFU where possible and an online tool^33^ was used to extract numerical data from figures. Comparisons between control and AMP-(intervention-) groups were made by calculating Hedge’s *g* and then obtaining the effect size to account for different measurement scales for the same unit and bias for small sample sizes (<20) using a correction factor.^34^ Values were then presented in a forest plot using RevMan5.4 (Review Manager (RevMan) free software, Version 5.4. Copenhagen: The Nordic Cochrane Centre, The Cochrane Collaboration). Continuous variables and a random-effects analysis model were used. The effect measure was standardized mean differences (SMD). An *I*^2^ value was also obtained to determine the heterogeneity between studies and relevant subgroups which further identifies the extent of variability between studies with a threshold of <60% for moderate or irrelevant heterogeneity and >70% as substantial heterogeneity.^35^ All calculations made followed the guidance of meta-analysis of data from animal studies by Vesterinen et al.^34^

## 3 Results

### 3.1 PRISMA Summary of Results

**Figure 1:**
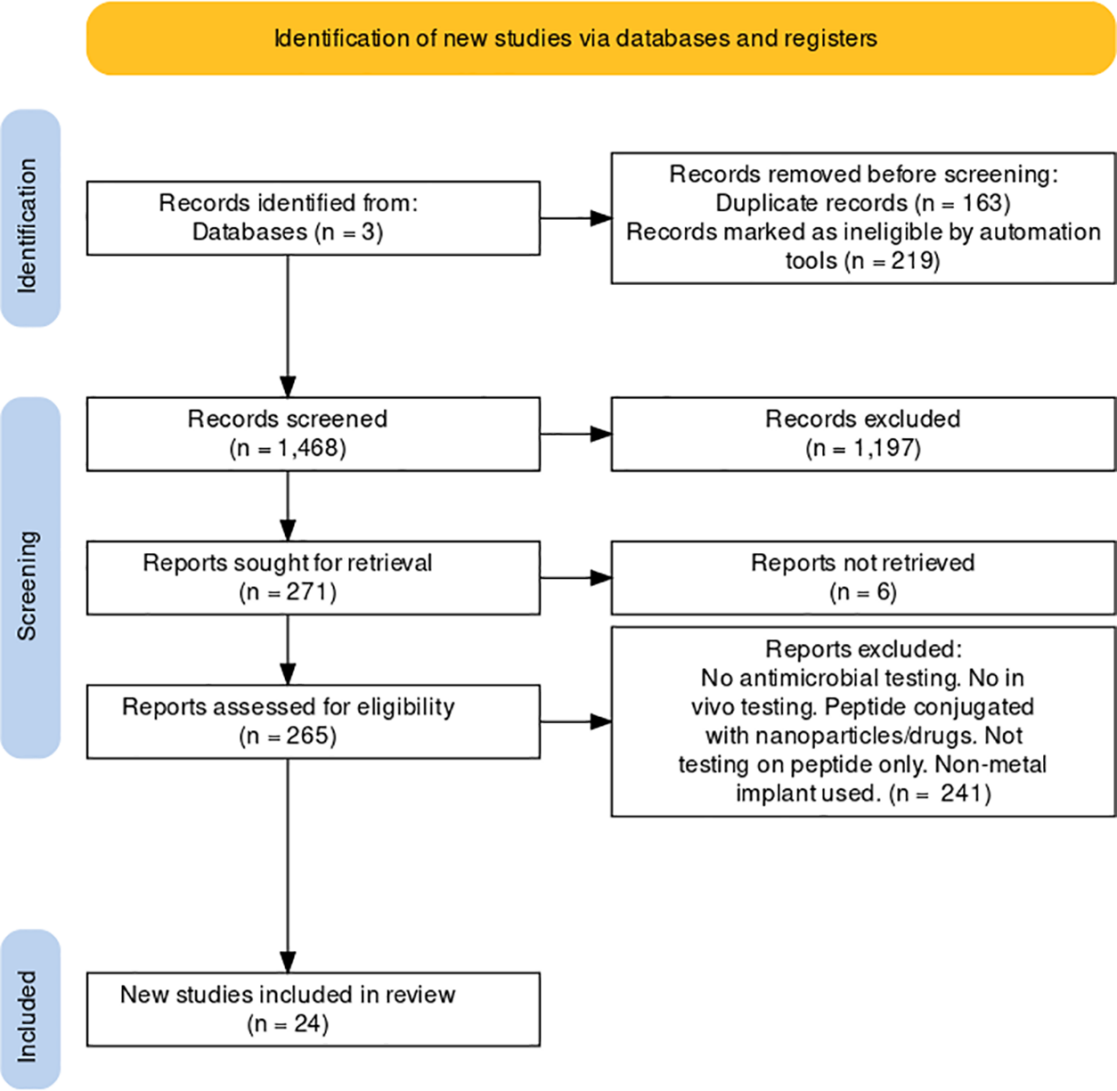
PRISMA framework. Representation of the steps taken to identify relevant studies for this review and the number of studies that remained after each step. The PRISMA flow diagram^31^ was adapted from a template on the PRISMA website (http://prisma-statement.org/prismastatement/flowdiagram.aspx).

Figure 1 shows the PRISMA results from the database search where a total of 1850 papers were found from three different databases. 1468 papers were then obtained after removal of duplicate (163) and review (219) papers. Further screening of abstracts resulted in exclusion of 1197 additional papers. Assessment of 265 retrieved full-text articles excluded 241 studies to remain with 24. Following this, 18 studies underwent a meta-analysis.

### 3.2 Characteristics of Included Studies

Following a database search and the steps from the PRISMA framework, a number of studies were selected based on specific criteria using a PICO model (Table 1). Table 2 shows the characteristics of included studies. 24 studies were screened for changes in bacterial counts from metal implants with and without AMPs where 19 different AMPs were identified. Six studies looked at VAN, five looked at HHC36, and the remaining AMPs were looked at individually by other studies. All studies demonstrated reduced CFU counts in comparison to their respective control except for DDDEEK^36^ which by itself had the same CFU counts as its respective control and RGD^37^ where the CFU counts were higher than the respective control. 22 out of 24 studies used titanium implants and 2 used stainless steel K-wires. The majority of the studies used *S. aureus* in vivo for the infection models.

**Table 2:** Characteristics of the 24 Included Studies

Study	AMP, surface type of implant, and animal type	Mechanisms of action of AMPs	Intervention and AMP binding method (modification method, bacteria strains)	Coating type	Outcome measures (Antibacterial test)	Results (Antibacterial effect)
Adams et al., 2009^38^	VAN; Ti rods; Male Wistar rats.	VAN blocks the construction of cell wall.	AMP dissolved in deionized water and added to sol–gel which was coated onto the wire and let dry for 2 h between each layer and 12 h after the last layer. Rat model of periprosthetic infection: 150 μL of 10^3^ CFU *Staphylococcus aureus* (SA) inoculated. AMP-containing sol–gel implant inserted or control coated rod.	Physical binding	Implants extracted, rolled over blood agar plates and incubated at 37 °C for 24 h. Rods sonicated and vortexed. Serially diluted samples plated onto agar plates at 37 °C for 24–48 h for CFU counts. Mean ± SE *n* = 3 (7, 14, 21 days) and 2 (21 days).	Day 7: 1.12 ± 0.52 for control and 3 ± 2.83 for AMP-group. Day 14: 2.19 ± 3 × 10^3^ for control group and 1.57 ± 1.14 for AMP group. Day 21: 1.78 ± 2.24 × 10^5^ and 1.3 ± 1.31 × 10^4^. 28 days: 1.88 ± 1.86 × 10^4^ control and 68 ± 71.2 for AMP group. All units CFU.
Li et al., 2009^39^	Interleukin 12 (IL-12); K-wires (stainless steel); Rat models.	IL-12 can influence T helper (Th) cells to secrete Th1 cytokines, instructing the cell-mediated immune system against bacterial invaders.	Implant immersed in negatively charged AMP solution and nanoscale coating achieved by electro-static (layer-by-layer) LBL self-assembly at implant/tissue interface. Open fracture rat model: fracture site accessed and ends revealed. 100 μL *S. aureus* (10^2^ CFU) injected. Fracture fixed after 1 h with K-wire.	Physical binding	Femurs homogenized and placed onto blood agar plates at 37 °C for 48 h. *n* = 12	21 days: Infection rates were 90.1% for control group, 100% for 0.5 ng group, 20% for 10.6 ng group, 40.2% for 21.2 ng group and 59.9% for 40.3 ng group.
Gao et al., 2011^40^	KRWRIRVRVIRKC (Tet-20); Ti surface; Female Sprague–Dawley rats.	The cationic peptide Tet-20 may act by inserting into the negatively charged bacterial cell membranes.	Ti modified with maleimide incubated with cysteine + AMP solution overnight. Rat infection model: incision made at dorsal side of rat and implants inserted. 250 μL *S. aureus* (10^8^) was injected. Control and AMP-coated groups.	Chemical binding	7 days: implants removed, placed in PBS solution and then sonicated for 10 min. Solutions serially diluted, plated and CFU counts taken. Mean ± SD, *N* = 14	7 days: at least 85% CFU decrease in 10 out of 14 rats and below 55% for the remaining 4.
Sinclair et al., 2013^41^	Cationic steroidal antimicrobial peptide-13 (CSA-13); Ti plug implant; Female Suffolk-cross sheep.	CSA-13 inserts into the negatively charged bacterial cell membranes and causes disruption of cellular integration.	CSA-13 coated regions of implant. Sheep: incision at distal joint of knee and medial metaphyseal flare of femur. Implants inserted and 200 μL Methicillin resistant *S. aureus* (5 × 10^6^ CFU) injected. Groups: contaminated porous-coated plug, 2 Ti-plug + contamination groups Ti-plug + contamination + Si polymer coating and uncontaminated Porous Ti implant without coating.	Physical binding	12 weeks: Culture swab of skin, subcutaneous, intramuscular and bone taken and streaked onto Columbia blood agar and incubated at 37 °C overnight. Tissue samples mixed with PBS, homogenized then sonicated. Serial dilutions performed and plated on TSA plates. Mean ± SD *N* = 3, 5	12 weeks: AMP-group had 23.3 ± 52.2 CFU versus control which had 1.14 ± 1.44 × 10^5^ CFU.
Windolf et al., 2014^42^	Lysostaphin; Ti discs; Female wild-type BALb/c mice.	Lysopstaphin can target sessile bacteria in a biofilm and directly destroys the extracellular biofilm matrix by cleavage of protein components.	Plates dipped into amino acid-based AMP-PDLLA solution and repeated twice for 10 μm coating thickness. Implant-associated bone infection model: bone defect from exposed Fascia and plate fixed to femur. 1 μL *S. aureus* (1.94 × 10^03^/μL) inoculated. Groups: AMP + PDLLA-coated, 1 mg/mL AMP + PDLLA-coated, coated Ti with 40 kGy-β irradiation, uncoated and PDLLA-only coated.	Physical binding	7, 14, and 28 days: CFU counts obtained. Bacteria taken by lavage from thighs where 200 μL serially diluted and 4 replicates plated on Columbia agar with 5% sheep blood. Plates were kept at 37 °C for 24 h and CFU/mL noted. Median values with whiskers (min/max). *n* = 10	Day 7: 6.32 × 10^2^ (min 5.29 × 10^1^; max 10^5^) control, 1.49 × 10^1^ and 1.25 × 10^2^ for AMP-group and 2.72 × 10^3^ for radiation group. Day 14: 9.44 × 10^1^ (min 0.978; max 3.76 × 10^4^) control, 2.30 and 2.95 × 10^1^ for AMP group and 2.76 × 10^2^ for radiation group. Day 21: 6.25 × 10^1^ (min 2.01; max 2.01 × 10^2^) control and 1.47 × 10^2^ for radiation group. No values for AMP-coated groups. All units CFU/mL.
Jennings et al., 2015^43^	VAN; Stainless steel wire (316L); Mice	See above	AMP solutions injected onto metal surface. Uncoated, coated + AMP delivery phosphatidyl-choline coated, AMP-coated and Amikacin coated. Catheter biofilm model: implanted into spine. *P. aeruginosa* (10^4^ CFU) + *S. aureus* (10^5^ CFU) inoculated.	Physical binding	2 days: 50 of the catheters and wires taken out and separated and analyzed by obtaining CFU counts and calculating clearance rates. Mean ± CI. *N* = 6	Day 2: Control groups had 2.52 ± 1.81 and 0.90 ± 1.23 for *S. aureus* and *P. aeruginosa*, respectively. 100% clearance for AMP groups against both strains. P group had 4 ± 1.3 with SA. P group had 1.24 ± 1.43, and A group had 0.14 ± 0.48 for *P. aeruginosa*. All other groups had 0. All units were log CFU.
Chen et al., 2016^44^	Cys-melimine (CTLISWIKNKRKQRPRVSRRRRRRGGRRRR) (Cys-Mel); Ti disks and Ti; Female BALB/c mice and male Sprague–Dawley rats.	Melimine can disrupt bacterial membranes, especially the integrity of the cytoplasmic membranes both for *P. aeruginosa* and *S. aureus.*	Amine functionalization on Ti surface and Cys-melimine attached using cross-linker solution into which the substrate was immersed. Subcutaneous rat and mouse model: incision at spine made down to the subdermal fascia. Disks or implants implanted, and wound was sealed. 100 μL *S. aureus* (10^5^ or 10^7^ CFU) injected into the area.	Chemical binding	5–7 days: Implants and surrounding tissue removed, immersed in PBS and vortexed or homogenized. CFU/disk counts taken after solutions underwent serial dilutions and plated in triplicate on nutrient agar. Incubation at 37° for 18 h. Mean ± SD *N* = 15 mice, 10 mice, 11 rats	5 days mice 10^7^: AMP-coated 1.75 ± 2.72 × 10^6^ and 5.35 ± 4.07 × 10^6^ for control. 5 days mice 10^5^: AMP-coated (1.6 ± 2.27 × 10^5^) and control (1.44 ± 1.12 × 10^6^). 7 days mice 10^5^: 2.42 ± 2.27 × 10^4^ AMP-coated and 3.26 ± 5.16 × 10^5^ control and for tissue 9.18 × 10^4^ ± 1.35 × 10^5^ AMP and 8.99 × 10^5^ ± 1.11 × 10^6^ control. 5 days rat 10^7^: control 2.17 ± 2.31 × 10^7^ and AMP 6.9 ± 8.03 × 10^5^ CFU/implant. 5 days rat 10^5^: 1.6 ± 1.54 × 10^7^ control and 3.14 ± 2.49 × 10^5^ AMP.
de Breij et al., 2016^45^	OP-145, (acetyl-IGKEFKRIVERIKRFLRELVRPLR-amide); TAN disks (Ti, aluminum and niobium); Female Charles River New Zealand white rabbits.	OP-145, a LL-37-derived synthetic peptide, can neutralize the bacterial toxins lipopolysaccharide (LPS) and lipoteichoic acid of *S. aureus*.	AMP-solution sprayed onto TAN nails. Rabbit intramedullary nail infection model: right humerus penetrated with a drill bit to access medullary cavity followed by lavage. 100 μL of *S. aureus* (6 × 10^4^ CFU) dropped followed by the insertion of the nail. Animal models had a control group and a coated group.	Physical binding	28 days rabbit: Surrounding tissue and humerus removed and homogenized. Nail extracted and sonicated. 10-fold serial dilutions plated on blood agar. Lower limit of detection 50 CFU/nail or/tissue and 100CFU/bone. Mean ± SD *n* = 7 and 6	Day 28: Control had 3.01 ± 5.97 × 10^5^ CFU/nail and AMP-group had 0.581 ± 1.3 × 10^5^ CFU/nail. Bone control had 0.736±1.02 × 10^7^ CFU/bone and 3.97 ± 8.85 × 10^6^ CFU/bone for AMP-group and for tissue, control group had 0.672 ± 1.34 × 10^6^ CFU and 2.29 ± 4.58 × 10^3^ CFU for AMP group.
Kucharíková et al., 2016^46^	VAN and/or Caspofungin (CAS); Ti implants; BALB/c female mice.	CAS (a lipopeptide) inhibits cell wall (1,3)-β-d-glucan synthesis in *Candida albicans*.	Peptides were covalently bound via silane-based Ti coating. Biomaterial-assisted murine model: Discs placed into subcutis and closed with surgical staples. 100 mL of *S. aureus* (bacteria) and *Candida albicans* (fungus) were inoculated 24h after surgery at 10^8^ cells/mL concentration.	Chemical binding	2 and 4 days: biomass quantification on discs. Surrounding tissue collected, sonicated and homogenized. Diluted suspensions plated on TSB and YPD agar. Plates incubated at 37 °C for 24 h (bacterial) and 48 h (fungal). CFU counts taken. Mean ± SEM *N* = 11 (control), 8 (VAN), 8 (control), 10 (CAS)	Day 2 (Fungal): Control group 4.58 ± 0.11 and CAS-Ti group 3.13 ± 0.31 log CFU/disc. 6.68 ± 0.15 control and 6.15 ± 0.23 log CFU/g tissue for Ti-Cas. Day 4 (Bacterial): Control group 5.26 ± 0.51 and VAN-Ti group had 2.97 ± 0.21 log CFU/disc. 7.50 ± 0.12 control and 7.51 ± 0.12 log CFU/g tissue for Ti-VAN group.
Nie et al., 2017^23^	Bacitracin, Ti_6_Al_4_V rods; female Sprague–Dawley rats	Bacitracin as a polypeptide antibiotic inhibits the formation of linear peptidoglycan chains, the main component of bacterial cell membranes.	Rods immersed in dopamine solution then in Bacitracin (1 mg/mL) dissolved in ethanoic acid at room temperature for 8 h. Rat osteomyelitis model: Contaminated Ti and Ti-AMP and uncontaminated Ti groups. 108 CFU/mL *S. aureus* inoculated. Bone cavity accessed and rods implanted.	Chemical binding	3 weeks: Bone tissue and femoral samples grounded, and rods sonicated after which serial dilutions were performed. Dilutions plated on agar overnight at 37 °C and CFU/g obtained. Mean ± SD *n* = 5	Day 21: Control group had 4.67 ± 2.34 × 10^5^ CFU and AMP-coated group had 3.13 ± 1.67 × 10^3^ CFU respectively. For bone tissue the control group had 2.89 ± 1.43 × 10^5^ CFU/g and 6.01 ± 3.23 × 10^3^ CFU/g for the AMP-coated group.
Stavrakis et al., 2019^47^	VAN; Ti K-wire; Male C57BL/6J mice.	See above	PEG–PSS polymer dissolved to have 20 mg/mL AMP. Wires immersed into solutions at 4 °C and dried at 50 °C 10 times. Control, PEG–PES + AMP groups. Wires implanted into defected femoral intramedullary canal. 2 μL SA Xen36 (10^8^ CFU) inoculated.	Chemical binding	42 days: Implant and surrounding tissue extracted, sonicated, and homogenized, respectively, and plated onto agar. CFU counts taken after 24 h later. Mean ±SEM *n* = 6	Day 42: control had 3.7 ± 1.1 × 10^5^ CFU and 2.8 ± 1.5 × 10^2^ from tissue and implant, respectively. VAN-coated pins had 2 ± 2 CFU in tissue samples. From implants, VAN-coated pins had 0 CFU.
Zhan et al., 2018^48^	HHC36 (KRWWKWWRR); Ti rods; New Zealand Albino rabbits.	HHC36 efficiently disrupts the bacterial membrane structure.	Ti rods treated and NIPAM polymerized to Ti-pNIPAM. HHC36 added by click chemistry; Ti-pNIPAM azidated to form reaction sites. Infection rabbit model: Tibia accessed and defect on medullary cavity created. Ti or Ti-pNIPAM-AMP samples implanted. Samples immersed in *S. aureus* (10^7^ CFU/mL) for 2 h at room temperature.	Chemical binding (click chemistry)	7 days: tibias removed and supernatant rolled over blood agar for semiquantification and antimicrobial activity. Bacteria detached and qualitative antibacterial activity measured after serial dilutions and using agar plates. *N* = 3	7 Days: 99.9% and 91.5% bacteria killed on the implant and surrounding tissue, respectively.
Zhang et al., 2018^8^	VAN; Ti implant; Female rabbits.	See above	Ti sprayed for hole through design and AMP incorporated. Groups: pure Ti coating, micropattern Ti coating with AMP and sterile Ti rod. Rabbit osteomyelitis model: rods contaminated with *S. aureus* (10^6^ CFU/mL) and implanted into left tibia of rabbits through the tibia plateau.	Physical binding	42 days: Samples and tibia (homogenized) removed and plated on pancreatic soy peptone agar and incubated at 37 °C for 24 h. CFU counts taken. Mean ± SD *N* = 10	Day 42: Control group had 8.42 ± 0.68 × 10^5^ CFU/Ti stick, and AMP-group had 4.04 ± 0.89 × 10^4^ CFU/Ti stick. Control values were 3.24 ± 0.38 × 10^4^ CFU/Tibia and AMP-group was 3.04 ± 0.37 × 10^3^.
Chen et al., 2019^49^	HHC36; Ti surface; New Zealand rabbits.	See above	Oxygen plasma treated Ti immersed in click AMP solution. Coated and uncoated Ti implants used. Rabbit osteomyelitis model: patellar ligament separated from left tibia and hole drilled. 50 μL of 5 × 10^6^ CFU *S. aureus* inoculated, and implants inserted.	Chemical binding (click chemistry)	7 days: implants and powdered tibia extracted and immersed in LB media. Bacterial solution diluted and plated on blood agar at 37 °C for 24 h. Mean ± SD *N* = 3	Day 7: Control groups from the implant and medullary cavity respectively had 8.5 ± 1.08 × 10^4^ CFU/cm^2^ and 1.16 × 10^4^ ± 10^6^ CFU/g. Peptide-coated samples had 1.83 ± 0.5 × 10^4^ CFU/cm^2^ and 0.36 ± 0.01 × 10^4^ CFU/g for implant and medullary cavity. Almost 100% bacteria on Ti-pNIPAM-AMP surface killed.
Gao et al., 2019^50^	Cationic peptide (cPep); TiO_2_ nanospike and Ti rods; Male Sprague–Dawley rats	The cationic peptide may act by inserting into the negatively charged bacterial cell membranes.	Ti rods underwent alkaline hydrothermal process for TiO_2_ nanospike coating and immersed in cPep solution. Rat model: subcutaneous implantation of 10 μL of *S. aureus* (10^8^ CFU) infected implants.	Physical binding	5 days: Implants removed and sonicated. After serial dilutions, samples plated on LB agar. Mean±SD *N* = 8	Day 5: Ti implants had 2.38 ± 1.99 × 10^8^ CFU and 1.32 ± 1.49 × 10^2^ CFU for coated.
Yang et al., 2019^36^	WRWRWR and DDDEEK; modified with G_4_-(DOPA)_4_; Ti_6_Al_4_V implants; Female Sprague–Dawley rats.	The cationic peptide may insert into the negatively charged bacterial cell membranes and cause disruption of cellular integration.	Immersion of implants in DGD or WGD water solutions. Rodent subcutaneous infection model: Lateral condyle of distal femur accessed by incision and screws inserted. 50 μL of *E. coli* or *S. aureus* 10^5^ CFU injected. Four groups: DGD or WGD infected with either *E. coli* or *S. aureus*.	Chemical binding	5 days: implants and surrounding tissues removed. Both were separated and homogenized then diluted where they were plated on agar plates with ampicillin. Mean ± SD *N* = 6	Day 5: Control & DGD screws 3.06 ± 0.92 × 10^4^ CFU and 3.49 ± 0.49 × 10^4^ CFU for *E. coli* and *S. aureus*. Surrounding tissue groups control and DGD-group had 1.07 ± 0.21 × 10^6^ and 1.27 ± 0.24 × 10^6^ CFU/mg tissue for *E. coli* and SA. WGD and WGD-DGD coated groups had no detectable bacteria.
Zhang et al., 2019^51^	Alkynylated VAN; Ti_6_Al_4_V pins; CL57BL/6 mice.	Modified Van inhibits cell wall synthesis.	Polymethacrylates grafted onto Ti alloy with azide-bearing side chains via surface-initiated atom transfer radical polymerization. Alkynylated AMP conjugated to their side chains via “click” reaction. Incision at knee, medial parapatellar arthrotomy, and intercondylar notch of femur exposed. Infection: Luria broth/Xen29 *S. aureus* solution (10^4^ CFU/mL) injected and insertion of pin.	Chemical binding (click chemistry)	21 days or 4 months: Pins extracted, put in 1 mL LB and vortexed for 5 min. Portions loaded onto P100 agar plates. Plates incubated at 37 °C for 12 h. CFU counts taken. *n* = 7	Day 21: Control group had 1485 ± 533 and 68 ± 71.2 CFU/pin for Ti-Van group.
Chen et al., 2020^52^	HHC36; Ti + TNT; Male New Zealand rabbits	See above	50 μL HHC36 added onto substrates. Rabbit osteomyelitis model: 40 μL *S. aureus* (10^8^ CFU/mL) injected, rods inserted and then sutured. Anodized groups: Ti, Ti-AMP, and Ti-PMMA-AMP.	Physical binding	7 days: tissues, implants and tibia removed and incubated in LB medium Mean ± SD *N* = 3	7 Days: 0.90 ± 0.3 × 10^8^ CFU/mL Ti-NTs group, 0.95 ± 1.8 × 10^5^ CFU/mL for Ti-NTs-A group and 0.97 ± 1.69 × 10^3^ CFU/mL on implants. In the medullary cavity, the Ti-NT group had 2.75 ± 0.61 × 10^8^ CFU/mL, Ti-NTs-A group had 1.95 ± 0.86 × 10^7^ CFU/mL and Ti-NTs-P-A group had 0.66 ± 0.75 × 10^7^ CFU/mL.
Xu et al., 2020^53^	E-poly-l-Lysine (EPL); Ti slides; Female Sprague–Dawley rats	EPL possesses broad antimicrobial spectrum against Gram-positive and Gram-negative bacteria.	AMP and catechol mixed and painted onto Ti plates after 3 days. Uncoated Ti and AMP-coated Ti slides preseeded in 10 μL Methicillin resistant *S. aureus* (10^7^ CFU/mL). Rats: incision made near rat spine where uncoated and coated samples implanted on either side.	Chemical Binding	5 days: Implants extracted, sonicated and serially diluted before plating on LB medium. CFU counts taken (mean ± SD). *n* = 13	Day 5: Control group had 3.61 ± 1.28 × 10^5^ CFU/implant and EPLC3 group had 3.24 ± 0.423 × 10^4^ CFU.
Chen et al., 2021^54^	Fusion peptide (FP) containing HHC36 and QK; Ti implant; New Zealand rabbits.	See above	AMP fusion with Lys-Lys and azido group added. Alkyl group added to Ti surface and incubated in click solutions using sodium borohydride reduction promoted CuAAC for FP coating. Bone defect model: holes drilled at femoral centerline and implants placed with 15 μL *S. aureus* (10^8^ CFU/mL). Control, Ti, Ti-125QK, Ti-125HHC36 and Ti-125FP groups.	Chemical binding	7 days: Implants and marrow placed in nutrient broth medium and shaken for 2 h at 37 °C. Solutions plated on agar and CFU counts taken. Mean ± SD *N* = 3	7 days: Ti group had 1.38 ± 0.18 × 10^5^ CFU, Ti-125QK group had 1.79 ± 0.68 × 10^5^ CFU, Ti-125AMP group had 6.67 ± 3.28 × 10^2^ CFU and the Ti-125FP group had 0.78 ± 0.51 × 10^3^ CFU.
Fang et al., 2021^37^	HHC36 and RGD; Ti surface; New Zealand Rabbits.	See above	AMPs dissolved in ethanol and Ti immersed for 4 h. Rabbit Bone defect model: Two holes perpendicular to femur centerline drilled. 15 μL *S. aureus* (7.5 × 10^6^ CFU) injected followed by implants. Ti, Ti–S, Ti-RGD, Ti-HHC36, and Ti-Dual groups.	Chemical binding (thiol–ene click chemistry)	7 days: Implants removed and placed in nutrient broth for 3 h at 37 °C and vortexed. Solution diluted in PBS and plated onto agar. Mean ±SD *N* = 3	7 days: Ti group had 76.8 ± 1.36 × 10^4^ CFU, Ti–S group had 8.01 ± 1.31 × 10^4^ CFU, Ti-RGD group had 8.09 × 10^4^ ± 7.97 × 10^3^ CFU and Ti HHC36 group had 1.27 × 10^3^ ± 4.15 × 10^2^ CFU.
Hwang et al., 2021^55^	NKC (APKAMKLLKKLLKLQKKGI) peptide; Ti; Sprague–Dawley male rats	The cationic peptide polymer may act by inserting into the negatively charged bacterial cell membranes.	AMP solution added to implants and incubated for 10 min at 37 °C. Rat subcutaneous infection model: incisions parallel to spine to subdermal fascia. Control and AMP-coated implants (Ti or PDMS) inserted at left or right respectively. 100 μL *P. aeruginosa* (2.5 × 10^8^ cells/mL) injected. Ti, PDMS, Ti-AMP, and Ti-PDMS groups.	Chemical binding (polydopamine chemistry)	5 days: Implants and surrounding tissue placed in PBS. Implants sonicated for 15 min and tissue samples homogenized before 10-fold serial dilutions. Solutions placed on LB agar and CFU counts taken. Mean ± SD *N* = 5	5 days: Ti control had 6.51 ± 5.16 × 10^3^ CFU/mL and AMP-coated had 14.6 ± 0.15 CFU/mL on implant surface. Ti control had 1.22 ± 1.25 × 10^6^ CFU/mL and AMP-coated had 4.04 ± 1.50 × 10^2^ CFU/mL from the surrounding tissue.
Yang et al., 2021^56^	Hyperbranched poly(l-lysine) (HBPL); Ti implants; Male Sprague–Dawley rats	The cationic peptide polymer may insert into the negatively charged bacterial cell membranes.	HBPL dissolved in water and implants immersed and incubated at 50° for 5 h for covalent grafting. Rat infection model: Incision made from metaphysis of tibial bone and bone marrow cavity drilled into. Screws inserted to reach bone cortex on the other side. 10 μL *S. aureus* (10^4^ CFU/mL) injected. Ti, Ti-GPTMS, and Ti-HBPL groups used.	Chemical binding	3 days: Tibias and screws removed, ultrasonicated in PBS solution and bacteria was diluted and plated on brain heart infusion agar. Plates cultured at 37 °C for 24 h and CFU counts taken. Mean ± SD *N* = 6	3 days: Control group had 3.45 ± 1.46 × 10^3^ and AMP group had 5.80 ± 3.93 × 10^2^ CFU.
Ye et al., 2021^57^	GL13K (AMP); Ti implants; Sprague–Dawley male rats.	The cationic peptide may insert into the negatively charged bacterial cell membranes.	AMP + deionized water vortexed. AMP self-assembly by addition of AMP solution into borax-NaOH with or without AgNP. Implant immersed into AMP solution overnight at room temperature. Subcutaneous infection model: incision parallel to spine made up to subdermal fascia and implants inserted. 100 μL MRSA (10^8^ CFU/mL) injected. Control, Ag, AMP, and AG + AMP groups.	Physical binding	4 days: Discs removed and immersed in PBS and surrounding tissue removed. Both samples were used for CFU counts. Mean ± SD, *N* = 10	4 days: Control group had 4.05 ± 2.80 × 10^7^ CFU and GL13K group had 9.84 × 10^6^ ± 1.14 × 10^7^ CFU.

### 3.3 Examples of Excluded Studies

Table 3 shows some examples of excluded studies from the full-text screening stage where studies contained most of the inclusion criteria but did not test for specific criteria. The table shows the study titles and the reasons for exclusion for studies that made it to the full-text eligibility stage of the PRISMA framework. For example, the study by Stewart et al.^58^ seemed to meet the criteria set out for this study, but the use of antibiotics immediately after surgery does not represent the AMP effects alone.

**Table 3:** Examples of Excluded Studies

Reference	Title	Reason for Exclusion
Alt et al., 2011^59^	Effects of gentamicin and gentamicin-RGD coatings on bone ingrowth and biocompatibility of cementless joint prostheses: an experimental study in rabbits	No artificial infection model induced in vivo
Stewart et al., 2012^58^	Vancomycin-modified implant surface inhibits biofilm formation and supports bone-healing in an infected osteotomy model in sheep: a proof-of-concept study	Antibiotic used immediately after surgery
Han et al., 2014^60^	BMP_2_-encapsulated chitosan coatings on functionalized Ti surfaces and their performance in vitro and in vivo	No artificial infection model induced in vivo. Penicillin injected for 3 days after surgery
Kucharíková et al., 2015^61^	In vivo *Candida glabrata* biofilm development on foreign bodies in a rat subcutaneous model	No AMP used
Shi et al., 2015^62^	Biological and immunotoxicity evaluation of antimicrobial peptide-loaded coatings using a layer-by-layer process on titanium	Effects on weight; no artificial induction model induced in vivo; AMP delivered via injection

### 3.4 Risk of Bias

The ROB tool from SYCRYL^32^ was used to determine the ROB in animal treatment within each study where Yes, No, and Unclear were appropriate responses and can be seen in Table S1. The majority of the responses in the table are “U”, which represent unclear. Eleven studies had animals housed randomly and only two studies had an allocation sequence where the responses for these categories were “Y” representing Yes as an answer and low bias for the specific question.

### 3.5 Effects of Intervention and Meta-Analysis

**Figure 2:**
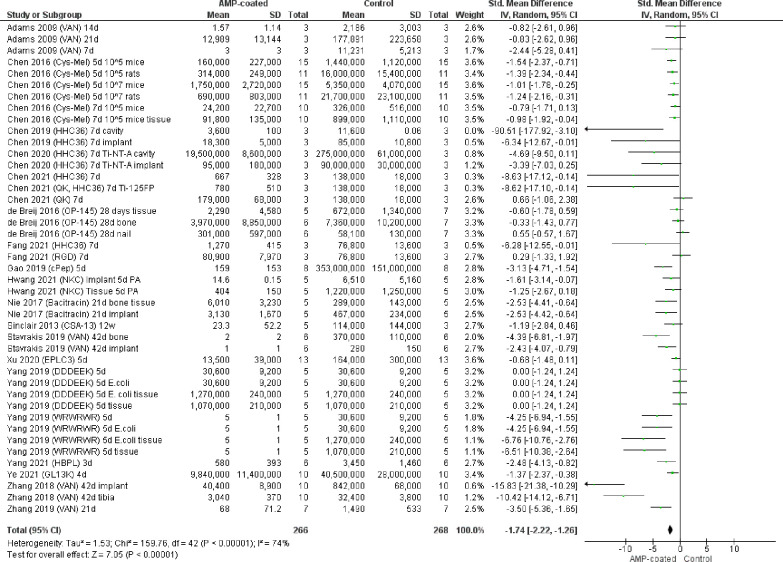
Forest plot of meta-analysis for assessment of AMP-coated implants for bacterial infection prevention as CFU counts expression. Mean, standard deviation (SD), and sample size taken from 18 studies to compare effect size using Hedge’s g as the meta-analysis. A forest plot plotted to show effect size comparing control and AMP-coated groups with 95% confidence intervals (CI) and overall effect size as a diamond. Vertical lines indicates no difference between the two groups. Heterogeneity between studies, τ^2^, χ^2^, and *I*^2^ were also calculated (*P* < 0.00001). IV, inverse variance. All bacteria used were *S. aureus* unless indicated otherwise (*E. coli* or *P. aeruginosa* (PA)).

From the 18 studies, the effect sizes were obtained from Hedges g. Figure 2, and Figures S1, S2, S3, and S4 show forest plots for an overall effect size and subgroup effect sizes. Subgroups compared were as follows: specific AMP used, animal species, duration of implantation, and sample location in Figures S1, S2, S3, and S4, respectively. Point 0 shows the line of no effect, and the total effect size appears as a diamond with 95% Confidence Interval (CI). It can be seen in Figure 2 that the studies used for meta-analysis are in favor of AMP-coating as they fall on the left of the line of no effect (SMD was −1.74, 95% CI [−2.26, −1.26], *p* < 0.00001). These studies comprised 534 animals in total, with 266 and 268 animals in the AMPs-coated and control groups, respectively. “Chen 2021 (QK) 7d”, “de Breij 2016 (OP145) 28d nail”, and “Fang 2021 (RGD) 7d” comparisons were on the right of the line of no effect in favor of the control. The DDDEEK comparisons did not favor AMP or control groups.

For each forest plot, significant effect sizes in favor of the AMP intervention are demonstrated except for the “DDDEEK”, “OP-145”, “Sheep”, “12 weeks”, “28 days”, and “14 days”, and subgroups due to the diamond touching the line of no effect. 74% Heterogeneity is seen from the *I*^2^ (*P* < 0.00001) between the studies in Figure 2. The *I*^2^ value represents the percentage of heterogeneity between studies or subgroups. The CI for the HHC36 is larger than those of the other comparisons (higher uncertainties).

Figure S1 produced *I*^2^ values of 86% for VAN (*p* < 0.00001) and 53% for “Other” (*p* < 0.02). HHC36 had 2% (*p* < 0.41), OP-145 had 6% (*p* < 0.35), and Melimine (*p* = 0.85), WRWRWR (*p* = 0.59) and DDDEEK (*p* = 1) all had 0% *I*^2^ values where the respective overall effect diamond touches the line of no effect. The “HHC36” and “OP-145” subgroups had low heterogeneity, while all other groups in Figure S1 had high heterogeneity. The between-subgroup heterogeneity was also high at 90.9% (*p* < 0.00001). Figure S2 had *I*^2^ values of 10% (*p* = 0.35), 78% (*p* < 0.00001), and 82% (*p* < 0.00001) for rat, mouse, and rabbit subgroups, respectively, demonstrating high heterogeneity for mouse and rabbit subgroups. Heterogeneity was low at 18.7% (*p* = 0.30). Figure S3 shows *I*^2^ values of 20% (*p* = 0.26) for 3–4 days, 68% for 5 days (*p* < 0.0001), 59% for 7 days (*p* < 0.005), 30% for 21 days (*p* = 0.23), 6% for 28 days (*p* = 0.35), and 91% for 42 days (*p* < 0.00001). 69.9% (*p* = 0.002) heterogeneity was seen between subgroups for Figure S3 which is high. Low heterogeneity can be seen in “3–4 days” and “21 day” subgroups where the other groups with values carry high heterogeneity. Figure S4 showed the implant and the surrounding tissue subgroups with high *I*^2^ values of 70% and 81%, respectively, (*p* < 0.00001) and 29% heterogeneity (*p* = 0.24) between subgroups. Subgroups were not eligible for heterogeneity when only one study was present in the subgroup as 0% heterogeneity was shown.

## 4 Discussion

The current study aimed to determine the benefits of AMP-coating on metal implants in in vivo infection models. The results demonstrate that studies identified in this review have proven that most of the AMPs are able to reduce bacterial numbers in their presence in comparison to their respective control. The meta-analysis further confirmed that the results from these studies are in favor of the AMP-coated implants. Heterogeneity values were mostly high overall and between most subgroups meaning that the difference in variance is not random. This could be due to factors other than the use of different AMPs that influence the effect size. HHC36 and OP-145 AMPs, rat models, and implantation for 3–4 days and 21 days within their respective subgroups demonstrated low heterogeneity suggesting random error as the cause for different variance.

The most common AMPs identified from the studies were VAN and HHC36. Among the lowest CFU counts were brought about by VAN, lysostaphin, HHC36, CSA-13, cPep, HBPL, and NKC indicating that these may be the most suitable AMPs for metal implant coating. Lowering the bacterial counts in significant amounts demonstrates the antibacterial capabilities especially since the induced infection models used higher numbers of bacteria for infection than would normally be expected.^47^ As a result, these may demonstrate the extent to which they are able to prevent infection.

Heterogeneities above 75% are considered high, indicating that differences between studies are due to factors other than the type of AMP used.^35^ From the meta-analyses, high heterogeneity from subgroup analysis may suggest the subgroups identified may not be responsible for the differences between studies. Such assumptions are not surprising, as the nature of this review did not limit the methods used in the studies. Overlapping of CIs further indicates that these differences are not random, but the high heterogeneities suggest otherwise.^63^ The forest plots may not show significant differences in effect size meaning a superior AMP would be difficult to identify but could suggest that AMPs may still ultimately have the desired beneficial effects. This implies that factors such as the coating method, microbial selection, animal model, and implant duration may be among the causes for the high heterogeneity and variance.^63^ The low heterogeneities obtained indicate random error between studies in their respective subgroups. It is key to note that some subgroups did contain information from the same study where more than one result were provided which may have affected the heterogeneity.

A common factor among most studies in this review is that AMP solutions were used to coat implants physically. Implants may need more than just physical coating but also should undergo refabrication and chemical modification to further ensure that AMPs are stably attached, and biofilms cannot form, and bacteria cannot survive.^40^ This may also be because implants require good in vivo biocompatibility such as bone repair materials.^39,64^ It has been suggested that such an approach does not guarantee sufficient coating densities and hence may affect the beneficial outcomes intended from AMP use.^51^ Positive effects of the AMPs from the meta-analysis, heterogeneity, and difference in effect sizes suggest additional factors are in play.

AMPs incorporation methods such as covalent immobilization can provide admirable drug densities where effects can last for up to 2 weeks.^51^ Covalent attachment of AMPs may be encouraged as it can potentially reduce cytotoxic effects from high concentrations of AMPs due to their uncontrolled release.^65^ This may be because of their nonspecific membrane specificity,^39^ molecular weight differences, or chemical structure. Covalent bonding can also encourage AMP stability by ensuring desired orientations for AMPs.^49,65^ Chen et al.^49^ found that HHC36 using polymer brush coating and “click” chemistry was able to reduce bacterial numbers in surrounding tissue as well. “Click” chemistry together with polymer brush coating may provide long-term activity against bacterial infection, although Zhang et al.^8^ found that was not the case with VAN. They found that although the AMP was successful on the implant surface, it was not mobile and thus was not able to have antimicrobial effects in adjacent areas. This suggested that the covalent incorporation of the AMPs may be a reason for higher CFU counts rather than the lack of AMP action itself. Chen et al.^49^ suggested that the lack of performance in surrounding areas may be due to proteolytic degradation of the peptides over 5 to 7 days in a biological medium where they claimed further research is needed. Zhang et al.^8^ found that VAN could only provide “short-range” protection due to its lack of mobility and was not able to perform at the endosteal bone surface after 21 days. These examples showed that AMP action is likely to be successful on the implant surface, and by the time they are needed in surrounding tissue, it is likely that the immune system will be able to help.^23^

Chen et al.^54^ argued that the use of only one AMP covalently bonded may help to keep infections away but may slow down osteointegration and wound healing. Based on this, the authors suggest the use of a fusion peptide comprising an AMP and another peptide to benefit healing and osteointegration. The use of fusion peptides may even result in antimicrobial effects for more strains. Interestingly, Zhan et al.^48^ used “click” chemistry to result in a temperature-sensitive approach where the AMP is exposed and therefore active against bacteria at room temperature and inactive at body temperature to reduce the possibility of toxicity. This was achieved using pNIPAM polymer. This approach can reduce the cytotoxic effects.^48^ Using mixed peptides may also be considered while retaining antimicrobial activity and biocompatibility, although Chen et al.^54^ advised that limited reaction sites on the implant surface may cancel out these benefits. These studies showed that covalent attachment of AMPs may be ideal for applications with metal implants but may not be the case for all implant types.

Gram-positive Staphylococci is mainly responsible for biomaterial-associated infection and more specifically, *S. aureus* (SA) and *S. epidermidis* while Gram-negative bacilli and enterococci are also able to result in infection.^7,51^ 34% of orthopedic implant-associated infections and osteomyelitis are due to infection with *S. aureus*.^66^ With consideration to the idea of mixed or fusion peptides, it is highly likely that it can be designed to tackle both Gram-positive and -negative bacterial strains. Fusion peptides on the implant surface do not take up as many reaction sites and thus increases the grafting density.^54,67^ With the focus of the identified studies being toward *S. aureus* in vivo, it may be plausible to consider targeting other strains as well. Additionally, there may be strains of *S. aureus* resistant to VAN or methicillin which may make it even harder to target.^41,68^ This may justify the use of *S. aureus* in most studies identified in this review. Some studies looked at *E. coli*,^36,48−50,52−54^*P. aeruginosa*,^43,44,52^*S. epidermidis*,^7^ and *Candida albicans*(46) in vivo. de Breij et al.^45^ found that testing the antimicrobial effects on different bacterial strains in vitro in defined conditions might not be able to produce the same effects in vivo. This indicates the need to test different bacterial strains, both Gram-negative and -positive in vivo.

Covalent immobilization of AMPs onto implant surfaces can also be done using a layer-by-layer (LBL) approach which Li et al.^39^ used IL-12 as their AMP and demonstrated antibacterial properties. This coating may mean the AMP is retained at the site of injury due to the ability of the nanoscale coating having controlled molecular structures and potentially prevent a burst release of AMPs. These layers mostly form electrostatic interactions, and with addition of drugs or AMPs hydrophobic, van der Waals and hydrogen bonding may become involved. When released, IL-12 has a short half-life in vivo which means it can also be degraded fast. It is important to note that this AMP works differently from others as this targets the immune system to activate macrophages where the direct effects on bacterial growth were not investigated by Li et al.^39^

The animals (mice, rats, rabbits, and sheep) used in these studies are relatively small in size which means that Kirshner wires and other devices that are small in size are not able to precisely match the porous coated Ti implant used in total joint replacement and spine procedures.^41,67^ This could suggest the animal models used are not able to fully represent AMP effects in humans. Furthermore, the duration of implantation in these animal models ranged from 1 day to 12 weeks which means comparisons between studies may not be reliable. Adams et al.^38^ in their artificial infection model in vivo used doses of bacteria higher than that found in an ideal clinical state and suggest the bone changes observed with periprosthetic infection in their study better represent AMP action in preserving bone. This would mean that the antimicrobial effects of AMPs are evident in bacterial removal, although wound healing and osteointegration would need to be investigated further for which implantation duration for longer time periods would be needed. Adams et al.^38^ noticed that with sol–gel, VAN was not released after 14 days in vivo. The authors argue that this should not be concerning, as the study used a higher number of bacteria than expected in an ideal situation and therefore should not bear clinical significance. Li et al.^39^ found that IL-12 release with a LBL approach was done over 9 days where O’Sullivan et al.^68^ claimed the first 10 days after traumatic injury are the most critical to remove bacteria. This suggested that the in vivo models may need to be observed for at least 10 days.

There are some study limitations that are to be considered. The AMPs proved effective in preventing bacterial infection in vivo, although this was only against limited strains. Additional research on the AMP effects on more bacterial strains or microbes in vivo would support their consideration for clinical trials. Each study followed a set of animal guidelines which was different to the others, increasing the chances of bias. This is because the standard ARRIVE guideline (Animal Research: Reporting of In Vivo Experiments) for animal studies has not been consistently followed.^69^ Following such guidelines will improve the in vivo implant studies and achieve more convincing results.

## 5 Conclusion

With the risks posed by biomaterial-associated infections, it is crucial to investigate approaches that prevent such infections. The AMPs identified in this systematic review demonstrated appropriate antibacterial efficacy when coating or incorporating the metal implants where bacterial counts determined their capabilities. Studies in this review have shown that AMPs are able to prevent bacterial growth and biofilm formation in vivo using artificial infection models. They have demonstrated that with further research into the AMP-incorporation methods, the number of different AMPs and the range of bacterial strains that can be targeted in vivo, the use of AMPs for coating metal implants may be suitable for clinical trials and further applications.

## Supporting Information Available

Table S1: SYCRYL Risk of Bias tool for included studies identified in this paper. Figure S1: Forest plot comparing AMP subgroups from meta-analysis using RevMan5.4. Figure S2: Forest plot comparing animal subgroups following meta-analysis using RevMan5.4. Figure S3: Forest plot comparing duration of implant in vivo from meta-analysis using RevMan5.4. Figure S4: Forest plot comparing the type of sample following implant removal by meta-analysis using RevMan5.4. (PDF)

## Abbreviations

AMPs: antimicrobial peptides
APTES: NH_2_-groups using (3-aminopropyl) triethoxysilane
ARRIVE: Animal Research: Reporting of In Vivo Experiments
CAS: Caspofungin
CFU: colony forming units
CI: confidence interval
CuAAC: copper-catalyzed azide–alkyne cycloaddition
DGD: DDDEEK + G_4_-(DOPA)_4_
DOPA: dopamine
LB: Luria Broth
LBL: Layer-by-Layer
HBPL: hyperbranched poly(l-lysine)
PDLLA: poly(d,l-lactic acid)
PDA: polydopamine
PEG–PSS: poly(ethylene glycol)-Bl-poly(propylene sulfide)
pNIPAM: poly(*N*-isopropylacrylamide)
PICO: population, intervention, comparison, and outcome
PRISMA: Preferred Reporting Items for Systematic Reviews and Meta-Analyses
rAMPS: ribosomal AMP
nrAMPs: nonribosomal AMPs
ROB: Risk of Bias
SD: standard deviation
SEM: standard error of mearns
SMD: standardized mean differences
SYRCLE: Systematic Review Centre for Laboratory Animal Experimentation
TAN: Ti, aluminum, and niobium
Ti: Titanium
TC–CH: Ti plate + Collagen I/HA
TC–CHH: Ti plate + Collagen I/HA/HACC
TSA: Tryptic Soy Agar
VAN: Vancomycin
TNT: titanium nanotubes
WGD: WRWRWR + G_4_-(DOPA)_4_; G_4_-(DOPA)_4_
