# Supplementary material for: In Vivo Antibacterial Efficacy of Antimicrobial Peptides Modified Metallic Implants—Systematic Review and Meta-Analysis
Source: ACS Biomater Sci Eng. 2022 Apr 12;8(5):1749–62. doi: 10.1021/acsbiomaterials.1c01307 (PMC9171719; doi:10.1021/acsbiomaterials.1c01307)

Supporting Information

In vivo antibacterial efficacy of antimicrobial peptides modified metallic implants – systematic review and meta-analysis

Amrit Kaur Sandhu, Ying Yang, Wen-Wu Li**

School of Pharmacy and Bioengineering, Keele University, Stoke-on-Trent, ST4 7QB, UK

*Corresponding authors:

Wen-Wu Li, w.li@keele.ac.uk;

Ying Yang, y.yang@keele.ac.uk

This document includes:

Table S1

Figures S1, S2, S3, S4

Table S1: SYCRYL Risk of Bias tool. Each study was given "Y", "N" or "U" to represent Yes, No or Unclear respectively. Yes, represents low bias, No, represents high bias and unclear shows that there is medium bias in the care of animals during studies.

**Study**	**Was the allocation sequence adequately generated and applied?**	**Were the groups similar at baseline or were they adjusted for confounders in the analysis?**	**Was the allocation adequately concealed?**	**Were the animals randomly housed during the experiment?**	**Were the caregivers and/or investigators blinded from knowledge which intervention each animal received during the experiment?**	**Were animals selected at random for outcome assessment?**	**Was the outcome assessor blinded?**	**Were incomplete outcome data adequately addressed?**	**Are reports of the study free of selective outcome reporting?**	**Was the study apparently free of other problems that could result in high risk of bias?**
**Adams et al., 2009**	U	Y	U	U	U	U	U	U	Y	Y
**Li et al., 2009**	U	Y	U	U	U	U	U	U	U	Y
**Gao et al., 2011**	U	Y	U	U	U	U	U	U	U	Y
**Sinclair et al., 2013**	U	Y	U	U	U	U	U	Y	Y	Y
**Windolf et al., 2014**	U	Y	U	U	U	U	U	U	Y	Y
**Jennings et al., 2015**	U	Y	U	U	U	U	U	U	U	Y
**Chen et al., 2016**	U	Y	U	Y	U	U	U	Y	Y	Y
**de Breij et al., 2016**	U	Y	U	Y	U	U	U	Y	Y	Y
**Kucharíková et al., 2016**	U	Y	U	Y	U	U	U	U	U	Y
**Nie et al., 2017**	U	Y	U	Y	U	U	U	U	U	Y
**Zhan et al., 2018**	Y	Y	U	Y	U	U	U	U	U	Y
**Zhang et al., 2018**	U	Y	U	Y	U	Y	U	Y	Y	Y
**Chen et al., 2019**	U	Y	U	U	U	U	U	U	U	Y
**Gao et al., 2019**	U	Y	U	Y	U	U	U	Y	Y	Y
**Stavrakis et al., 2019**	U	Y	U	Y	Y	U	Y	Y	Y	Y
**Yang et al., 2019**	U	Y	U	U	U	U	U	U	U	Y
**Zhang et al., 2019**	U	Y	U	U	U	U	U	U	Y	Y
**Chen et al., 2020**	U	Y	U	U	U	U	U	U	U	Y
**Xu et al., 2020**	U	Y	U	Y	U	U	U	U	U	Y
**Chen et al., 2021** [47]	U	Y	U	U	U	U	U	U	U	Y
**Fang et al., 2021**	U	Y	U	U	U	U	U	U	U	Y
**Hwang et al., 2021**	U	Y	U	Y	U	U	U	U	U	Y
**Yang et al., 2021**	Y	Y	U	Y	U	Y	U	U	U	Y
**Ye et al., 2021**	U	Y	U	U	U	U	U	U	U	Y

Figure S1: Forest plot of subgroup meta-analysis for assessment of AMP-coated implants bacterial infection prevention as CFU counts expression, considering AMP subgroups. Mean, standard deviation & sample size from 18 studies comparing subgroup effect size (forest plot) in control and AMP-coated groups using Hedge's g as the meta-analysis, 95% Confidence Intervals and overall effect size as a diamond. Heterogeneity, Tau^2, Chi^2 and I^2 also calculated (P<0.00001). IV, inverse variance. All bacteria used were *S. aureus* unless indicated otherwise (*E. coli* or *P. aeruginosa* (PA)).

	AMP-coated			Control				Std. Mean Difference
Study or Subgroup	Mean	SD	Total	Mean	SD	Total	Weight	IV, Random, 95% CI
**1.2.1 Vancomycin**								
Adams 2009 (VAN) 14d	1.57	1.14	3	2,186	3,003	3	2.6%	-0.82 [-2.61, 0.96]
Adams 2009 (VAN) 21d	12,989	13,144	3	177,891	223,658	3	2.6%	-0.83 [-2.62, 0.96]
Adams 2009 (VAN) 7d	3	3	3	11,231	5,213	3	1.7%	-2.44 [-5.28, 0.41]
Stavrakis 2019 (VAN) 42d bone	2	2	6	370,000	110,000	6	2.0%	-4.39 [-6.81, -1.97]
Stavrakis 2019 (VAN) 42d implant	1	1	6	280	150	6	2.7%	-2.43 [-4.07, -0.79]
Zhang 2018 (VAN) 42d implant	40,400	8,900	10	842,000	68,000	10	0.6%	-15.83 [-21.38, -10.29]
Zhang 2018 (VAN) 42d tibia	3,040	370	10	32,400	3,800	10	1.2%	-10.42 [-14.12, -6.71]
Zhang 2019 (VAN) 21d	68	71.2	7	1,490	533	7	2.5%	-3.50 [-5.36, -1.65]
**Subtotal (95% CI)**			**48**			**48**	**15.9%**	**-4.27 [-6.43, -2.11]**
Heterogeneity: Tau² = 7.80; Chi² = 49.23, df = 7 (P < 0.00001); I² = 86%								
Test for overall effect: Z = 3.87 (P = 0.0001)								
**1.2.2 HHC36**								
Chen 2019 (HHC36) 7d cavity	3,600	100	3	11,600	0.06	3	0.0%	-90.51 [-177.92, -3.10]
Chen 2019 (HHC36) 7d implant	18,300	5,000	3	85,000	10,800	3	0.5%	-6.34 [-12.67, -0.01]
Chen 2020 (HHC36) 7d Ti-NT-A cavity	19,500,000	8,600,000	3	275,000,000	61,000,000	3	0.8%	-4.69 [-9.50, 0.11]
Chen 2020 (HHC36) 7d Ti-NT-A implant	95,000	180,000	3	90,000,000	30,000,000	3	1.2%	-3.39 [-7.03, 0.25]
Chen 2021 (HHC36) 7d	667	328	3	138,000	18,000	3	0.3%	-8.63 [-17.12, -0.14]
Chen 2021 (QK, HHC36) 7d Ti-125FP	780	510	3	138,000	18,000	3	0.3%	-8.62 [-17.10, -0.14]
Fang 2021 (HHC36) 7d	1,270	415	3	76,800	13,600	3	0.5%	-6.28 [-12.55, -0.01]
**Subtotal (95% CI)**			**21**			**21**	**3.7%**	**-5.26 [-7.56, -2.96]**
Heterogeneity: Tau² = 0.24; Chi² = 6.14, df = 6 (P = 0.41); I² = 2%								
Test for overall effect: Z = 4.48 (P < 0.00001)								
**1.2.3 Melimine**								
Chen 2016 (Cys-Mel) 5d 10^5 mice	160,000	227,000	15	1,440,000	1,120,000	15	3.6%	-1.54 [-2.37, -0.71]
Chen 2016 (Cys-Mel) 5d 10^5 rats	314,000	249,000	11	16,000,000	15,400,000	11	3.4%	-1.39 [-2.34, -0.44]
Chen 2016 (Cys-Mel) 5d 10^7 mice	1,750,000	2,720,000	15	5,350,000	4,070,000	15	3.6%	-1.01 [-1.78, -0.25]
Chen 2016 (Cys-Mel) 5d 10^7 rats	690,000	803,000	11	21,700,000	23,100,000	11	3.5%	-1.24 [-2.16, -0.31]
Chen 2016 (Cys-Mel) 7d 10^5 mice	24,200	22,700	10	326,000	516,000	10	3.5%	-0.79 [-1.71, 0.13]
Chen 2016 (Cys-Mel) 7d 10^5 mice tissue	91,800	135,000	10	899,000	1,110,000	10	3.5%	-0.98 [-1.92, -0.04]
**Subtotal (95% CI)**			**72**			**72**	**21.0%**	**-1.16 [-1.52, -0.80]**
Heterogeneity: Tau² = 0.00; Chi² = 1.96, df = 5 (P = 0.85); I² = 0%								
Test for overall effect: Z = 6.33 (P < 0.00001)								
**1.2.4 WRWRWR**								
Yang 2019 (WRWRWR) 5d	5	1	5	30,600	9,200	5	1.8%	-4.25 [-6.94, -1.55]
Yang 2019 (WRWRWR) 5d E.coli	5	1	5	30,600	9,200	5	1.8%	-4.25 [-6.94, -1.55]
Yang 2019 (WRWRWR) 5d E.coli tissue	5	1	5	1,270,000	240,000	5	1.1%	-6.76 [-10.76, -2.76]
Yang 2019 (WRWRWR) 5d tissue	5	1	5	1,070,000	210,000	5	1.1%	-6.51 [-10.38, -2.64]
**Subtotal (95% CI)**			**20**			**20**	**5.8%**	**-5.01 [-6.58, -3.44]**
Heterogeneity: Tau² = 0.00; Chi² = 1.93, df = 3 (P = 0.59); I² = 0%								
Test for overall effect: Z = 6.25 (P < 0.00001)								
**1.2.6 DDDEEK**								
Yang 2019 (DDDEEK) 5d	30,600	9,200	5	30,600	9,200	5	3.2%	0.00 [-1.24, 1.24]
Yang 2019 (DDDEEK) 5d E.coli	30,600	9,200	5	30,600	9,200	5	3.2%	0.00 [-1.24, 1.24]
Yang 2019 (DDDEEK) 5d E. coli tissue	1,270,000	240,000	5	1,270,000	240,000	5	3.2%	0.00 [-1.24, 1.24]
Yang 2019 (DDDEEK) 5d tissue	1,070,000	210,000	5	1,070,000	210,000	5	3.2%	0.00 [-1.24, 1.24]
**Subtotal (95% CI)**			**20**			**20**	**12.6%**	**0.00 [-0.62, 0.62]**
Heterogeneity: Tau² = 0.00; Chi² = 0.00, df = 3 (P = 1.00); I² = 0%								
Test for overall effect: Z = 0.00 (P = 1.00)								
**1.2.7 OP-145**								
de Breij 2016 (OP-145) 28 days tissue	2,290	4,580	5	672,000	1,340,000	7	3.2%	-0.60 [-1.78, 0.59]
de Breij 2016 (OP-145) 28d bone	3,970,000	8,850,000	6	7,360,000	10,200,000	7	3.3%	-0.33 [-1.43, 0.77]
de Breij 2016 (OP-145) 28d nail	301,000	597,000	6	58,100	130,000	7	3.3%	0.55 [-0.57, 1.67]
**Subtotal (95% CI)**			**17**			**21**	**9.8%**	**-0.11 [-0.78, 0.56]**
Heterogeneity: Tau² = 0.02; Chi² = 2.12, df = 2 (P = 0.35); I² = 6%								
Test for overall effect: Z = 0.33 (P = 0.74)								
**1.2.8 Other**								
Chen 2021 (QK) 7d	179,000	68,000	3	138,000	18,000	3	2.6%	0.66 [-1.06, 2.38]
Fang 2021 (RGD) 7d	80,900	7,970	3	76,800	13,600	3	2.7%	0.29 [-1.33, 1.92]
Gao 2019 (cPep) 5d	159	153	8	353,000,000	151,000,000	8	2.8%	-3.13 [-4.71, -1.54]
Hwang 2021 (NKC) Implant 5d PA	14.6	0.15	5	6,510	5,160	5	2.8%	-1.61 [-3.14, -0.07]
Hwang 2021 (NKC) Tissue 5d PA	404	150	5	1,220,000	1,250,000	5	3.0%	-1.25 [-2.67, 0.18]
Nie 2017 (Bacitracin) 21d bone tissue	6,010	3,230	5	289,000	143,000	5	2.5%	-2.53 [-4.41, -0.64]
Nie 2017 (Bacitracin) 21d implant	3,130	1,670	5	467,000	234,000	5	2.5%	-2.53 [-4.42, -0.64]
Sinclair 2013 (CSA-13) 12w	23.3	52.2	5	114,000	144,000	3	2.7%	-1.19 [-2.84, 0.46]
Xu 2020 (EPLC3) 5d	13,500	39,000	13	164,000	300,000	13	3.6%	-0.68 [-1.48, 0.11]
Yang 2021 (HBPL) 3d	580	393	6	3,450	1,460	6	2.7%	-2.48 [-4.13, -0.82]
Ye 2021 (GL13K) 4d	9,840,000	11,400,000	10	40,500,000	28,000,000	10	3.4%	-1.37 [-2.37, -0.38]
**Subtotal (95% CI)**			**68**			**66**	**31.3%**	**-1.37 [-2.01, -0.74]**
Heterogeneity: Tau² = 0.58; Chi² = 21.44, df = 10 (P = 0.02); I² = 53%								
Test for overall effect: Z = 4.22 (P < 0.0001)								
**Total (95% CI)**			**266**			**268**	**100.0%**	**-1.74 [-2.22, -1.26]**
Heterogeneity: Tau² = 1.53; Chi² = 159.76, df = 42 (P < 0.00001); I² = 74%								
Test for overall effect: Z = 7.05 (P < 0.00001)								
Test for subgroup differences: Chi² = 65.58, df = 6 (P < 0.00001), I² = 90.9%								

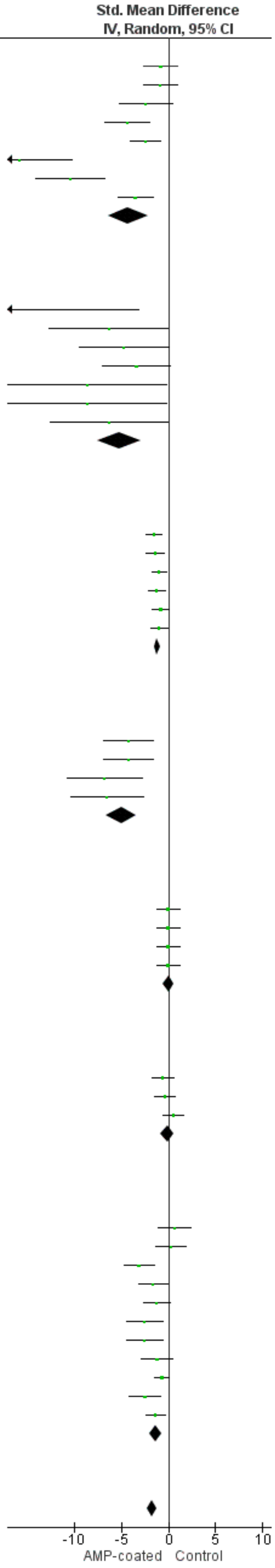

Figure S2: Forest plot of subgroup meta-analysis for assessment of AMP-coated implants on bacterial infection prevention as CFU counts expression, considering animal subgroups. Mean, standard deviation and sample size taken from 18 studies comparing effect size using Hedge's g as the meta-analysis. Effect size shown comparing control and AMP-coated groups with 95% Confidence Intervals and overall effect size as a diamond. Heterogeneity between studies, Tau^2, Chi^2 and I^2 were also calculated (P<0.00001). Subgroup analysis further compares overall effects sizes for different animal models used. IV, inverse variance. All bacteria used were *S. aureus* unless indicated otherwise (*E. coli* or *P. aeruginosa* (PA)).

Study or Subgroup	AMP-coated Mean	AMP-coated SD	AMP-coated Total	Control Mean	Control SD	Control Total	Weight	Std. Mean Difference IV, Random, 95% CI
**1.3.1 Rat**								
Adams 2009 (VAN) 14d	1.57	1.14	3	2,186	3,003	3	2.6%	-0.82 [-2.61, 0.96]
Adams 2009 (VAN) 21d	12,989	13,144	3	177,891	223,657	3	2.6%	-0.83 [-2.62, 0.96]
Adams 2009 (VAN) 7d	3	3	3	11,231	5,213	3	1.7%	-2.44 [-5.28, 0.41]
Chen 2016 (Cys-Mel) 5d 10^5 rats	314,000	249,000	11	16,000,000	15,400,000	11	3.4%	-1.39 [-2.34, -0.44]
Chen 2016 (Cys-Mel) 5d 10^7 rats	690,000	803,000	11	21,700,000	23,100,000	11	3.5%	-1.24 [-2.16, -0.31]
Gao 2019 (cPep) 5d	159	153	8	353,000,000	151,000,000	8	2.8%	-3.13 [-4.71, -1.54]
Hwang 2021 (NKC) Implant 5d PA	14.6	0.15	5	6,510	5,160	5	2.8%	-1.61 [-3.14, -0.07]
Hwang 2021 (NKC) Tissue 5d PA	404	150	5	1,220,000	1,250,000	5	3.0%	-1.25 [-2.67, 0.18]
Nie 2017 (Bacitracin) 21d implant	3,130	1,670	5	467,000	234,000	5	2.5%	-2.53 [-4.42, -0.64]
Xu 2020 (EPLC3) 5d	13,500	39,000	13	164,000	300,000	13	3.6%	-0.68 [-1.48, 0.11]
Yang 2021 (HBPL) 3d	580	393	6	3,450	1,460	6	2.7%	-2.48 [-4.13, -0.82]
Ye 2021 (GL13K) 4d	9,840,000	11,400,000	10	40,500,000	28,000,000	10	3.4%	-1.37 [-2.37, -0.38]
**Subtotal (95% CI)**			**83**			**83**	**34.5%**	**-1.41 [-1.80, -1.01]**
Heterogeneity: Tau² = 0.05; Chi² = 12.21, df = 11 (P = 0.35); I² = 10%								
Test for overall effect: Z = 7.02 (P < 0.00001)								
**1.3.2 Mouse**								
Chen 2016 (Cys-Mel) 5d 10^5 mice	160,000	227,000	15	1,440,000	1,120,000	15	3.6%	-1.54 [-2.37, -0.71]
Chen 2016 (Cys-Mel) 5d 10^7 mice	1,750,000	2,720,000	15	5,350,000	4,070,000	15	3.6%	-1.01 [-1.78, -0.25]
Chen 2016 (Cys-Mel) 7d 10^5 mice	24,200	22,700	10	326,000	516,000	10	3.5%	-0.79 [-1.71, 0.13]
Chen 2016 (Cys-Mel) 7d 10^5 mice tissue	91,800	135,000	10	899,000	1,110,000	10	3.5%	-0.98 [-1.92, -0.04]
Chen 2019 (HHC36) 7d cavity	3,600	100	3	11,600	0.06	3	0.0%	-90.51 [-177.92, -3.10]
Chen 2020 (HHC36) 7d Ti-NT-A cavity	19,500,000	8,600,000	3	275,000,000	61,000,000	3	0.8%	-4.69 [-9.50, 0.11]
de Breij 2016 (OP-145) 28 days tissue	2,290	4,580	5	672,000	1,340,000	7	3.2%	-0.60 [-1.78, 0.59]
de Breij 2016 (OP-145) 28d bone	3,970,000	8,850,000	6	7,360,000	10,200,000	7	3.3%	-0.33 [-1.43, 0.77]
Nie 2017 (Bacitracin) 21d bone tissue	6,010	3,230	5	289,000	143,000	5	2.5%	-2.53 [-4.41, -0.64]
Stavrakis 2019 (VAN) 42d bone	2	2	6	370,000	110,000	6	2.0%	-4.39 [-6.81, -1.97]
Stavrakis 2019 (VAN) 42d implant	1	1	6	280	150	6	2.7%	-2.43 [-4.07, -0.79]
Yang 2019 (DDDEEK) 5d E. coli tissue	1,270,000	240,000	5	1,270,000	240,000	5	3.2%	0.00 [-1.24, 1.24]
Yang 2019 (DDDEEK) 5d tissue	1,070,000	210,000	5	1,070,000	210,000	5	3.2%	0.00 [-1.24, 1.24]
Yang 2019 (WRWRWR) 5d E.coli tissue	5	1	5	1,270,000	240,000	5	1.1%	-6.76 [-10.76, -2.76]
Yang 2019 (WRWRWR) 5d tissue	5	1	5	1,070,000	210,000	5	1.1%	-6.51 [-10.38, -2.64]
Zhang 2018 (VAN) 42d tibia	3,040	370	10	32,400	3,800	10	1.2%	-10.42 [-14.12, -6.71]
Zhang 2019 (VAN) 21d	68	71.2	7	1,490	533	7	2.5%	-3.50 [-5.36, -1.65]
**Subtotal (95% CI)**			**121**			**124**	**40.8%**	**-2.00 [-2.79, -1.21]**
Heterogeneity: Tau² = 1.73; Chi² = 73.49, df = 16 (P < 0.00001); I² = 78%								
Test for overall effect: Z = 4.95 (P < 0.00001)								
**1.3.3 Rabbit**								
Chen 2019 (HHC36) 7d implant	18,300	5,000	3	85,000	10,800	3	0.5%	-6.34 [-12.67, -0.01]
Chen 2020 (HHC36) 7d Ti-NT-A implant	95,000	180,000	3	90,000,000	30,000,000	3	1.2%	-3.39 [-7.03, 0.25]
Chen 2021 (HHC36) 7d	667	328	3	138,000	18,000	3	0.3%	-8.63 [-17.12, -0.14]
Chen 2021 (QK, HHC36) 7d Ti-125FP	780	510	3	138,000	18,000	3	0.3%	-8.62 [-17.10, -0.14]
Chen 2021 (QK) 7d	179,000	68,000	3	138,000	18,000	3	2.6%	0.66 [-1.06, 2.38]
de Breij 2016 (OP-145) 28d nail	301,000	597,000	6	58,100	130,000	7	3.3%	0.55 [-0.57, 1.67]
Fang 2021 (HHC36) 7d	1,270	415	3	76,800	13,600	3	0.5%	-6.28 [-12.55, -0.01]
Fang 2021 (RGD) 7d	80,900	7,970	3	76,800	13,600	3	2.7%	0.29 [-1.33, 1.92]
Yang 2019 (DDDEEK) 5d	30,600	9,200	5	30,600	9,200	5	3.2%	0.00 [-1.24, 1.24]
Yang 2019 (DDDEEK) 5d E.coli	30,600	9,200	5	30,600	9,200	5	3.2%	0.00 [-1.24, 1.24]
Yang 2019 (WRWRWR) 5d	5	1	5	30,600	9,200	5	1.8%	-4.25 [-6.94, -1.55]
Yang 2019 (WRWRWR) 5d E.coli	5	1	5	30,600	9,200	5	1.8%	-4.25 [-6.94, -1.55]
Zhang 2018 (VAN) 42d implant	40,400	8,900	10	842,000	68,000	10	0.6%	-15.83 [-21.38, -10.29]
**Subtotal (95% CI)**			**57**			**58**	**22.0%**	**-2.62 [-4.20, -1.04]**
Heterogeneity: Tau² = 5.29; Chi² = 67.50, df = 12 (P < 0.00001); I² = 82%								
Test for overall effect: Z = 3.25 (P = 0.001)								
**1.3.4 Sheep**								
Sinclair 2013 (CSA-13) 12w	23.3	52.2	5	114,000	144,000	3	2.7%	-1.19 [-2.84, 0.46]
**Subtotal (95% CI)**			**5**			**3**	**2.7%**	**-1.19 [-2.84, 0.46]**
Heterogeneity: Not applicable								
Test for overall effect: Z = 1.42 (P = 0.16)								
**Total (95% CI)**			**266**			**268**	**100.0%**	**-1.74 [-2.22, -1.26]**
Heterogeneity: Tau² = 1.53; Chi² = 159.76, df = 42 (P < 0.00001); I² = 74%								
Test for overall effect: Z = 7.05 (P < 0.00001)								
Test for subgroup differences: Chi² = 3.69, df = 3 (P = 0.30), I² = 18.7%								

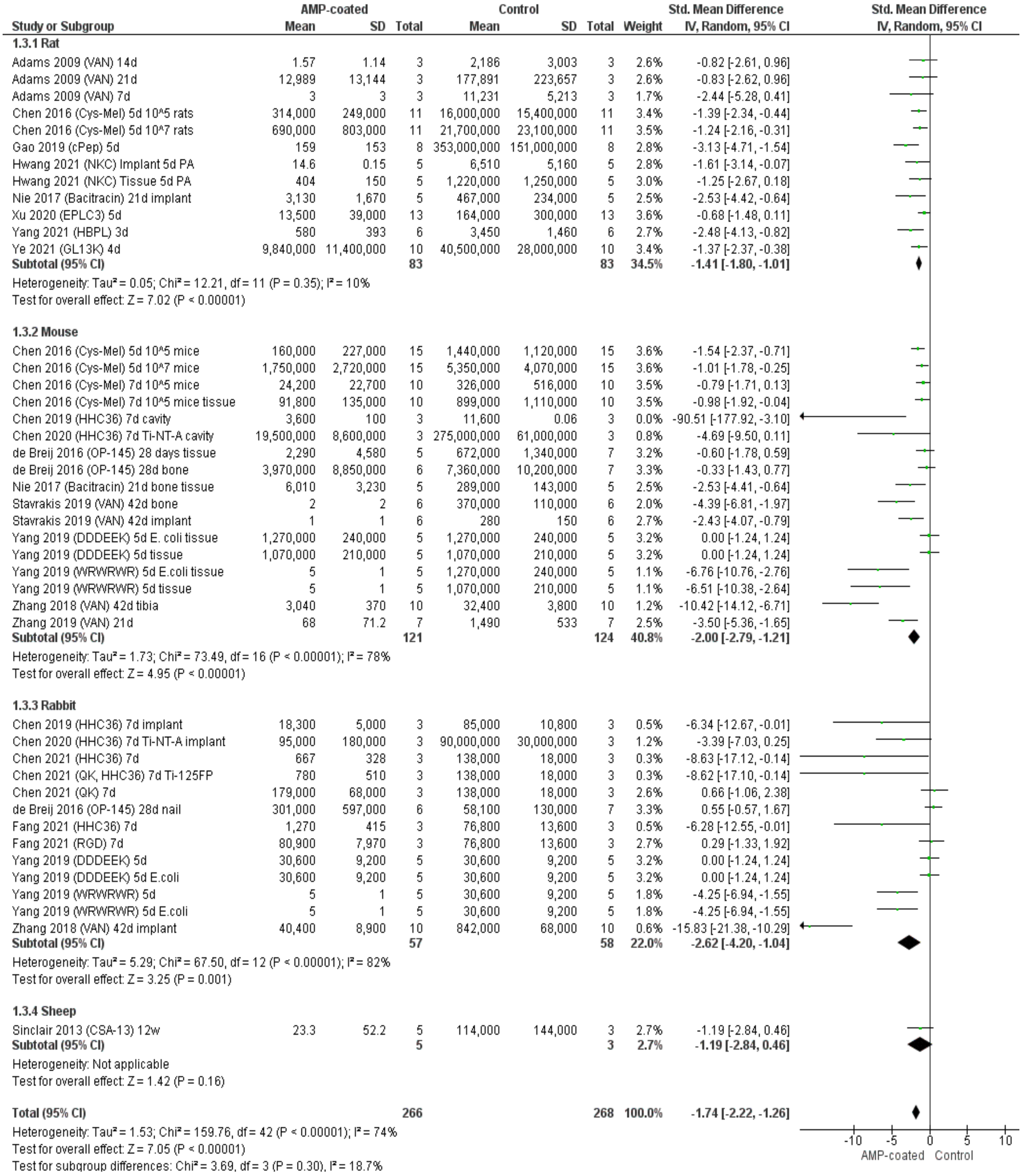

Figure S3: Forest plot of subgroup metal-analysis for assessment of AMP-coated implants on bacterial infection prevention as CFU counts expression, considering implant duration. Mean, SD and sample size taken from 18 studies to compare effect size using Hedge's g as the meta-analysis. A forest plot plotted to show effect size comparing control and AMP-coated groups with 95% Confidence Intervals and overall effect size as a diamond. Heterogeneity between studies, Tau^2, Chi^2 and I^2 were also calculated (P<0.00001). Subgroup analysis further compares overall effects sizes for implant duration in vivo. IV, inverse variance. All bacteria used were S *S. aureus* unless indicated otherwise (*E. coli* or *P. aeruginosa* (PA)).

	AMP-coated			Control				Std. Mean Difference
**Study or Subgroup**	**Mean**	**SD**	**Total**	**Mean**	**SD**	**Total**	**Weight**	**IV, Random, 95% CI**
**1.4.1 3-4 Days**								
Yang 2021 (HBPL) 3d	580	393	6	3,450	1,460	6	2.7%	-2.48 [-4.13, -0.82]
Ye 2021 (GL13K) 4d	9,840,000	11,400,000	10	40,500,000	28,000,000	10	3.4%	-1.37 [-2.37, -0.38]
**Subtotal (95% CI)**			**16**			**16**	**6.1%**	**-1.72 [-2.72, -0.72]**
Heterogeneity: Tau² = 0.12; Chi² = 1.25, df = 1 (P = 0.26); I² = 20%								
Test for overall effect: Z = 3.36 (P = 0.0008)								
**1.4.2 5 Days**								
Chen 2016 (Cys-Mel) 5d 10^5 mice	160,000	227,000	15	1,440,000	1,120,000	15	3.6%	-1.54 [-2.37, -0.71]
Chen 2016 (Cys-Mel) 5d 10^5 rats	314,000	249,000	11	16,000,000	15,400,000	11	3.4%	-1.39 [-2.34, -0.44]
Chen 2016 (Cys-Mel) 5d 10^7 mice	1,750,000	2,720,000	15	5,350,000	4,070,000	15	3.6%	-1.01 [-1.78, -0.25]
Chen 2016 (Cys-Mel) 5d 10^7 rats	690,000	803,000	11	21,700,000	23,100,000	11	3.5%	-1.24 [-2.16, -0.31]
Gao 2019 (cPep) 5d	159	153	8	353,000,000	151,000,000	8	2.8%	-3.13 [-4.71, -1.54]
Hwang 2021 (NKC) Implant 5d PA	14.6	0.15	5	6,510	5,160	5	2.8%	-1.61 [-3.14, -0.07]
Hwang 2021 (NKC) Tissue 5d PA	404	150	5	1,220,000	1,250,000	5	3.0%	-1.25 [-2.67, 0.18]
Xu 2020 (EPLC3) 5d	13,500	39,000	13	164,000	300,000	13	3.6%	-0.68 [-1.48, 0.11]
Yang 2019 (DDDEEK) 5d	30,600	9,200	5	30,600	9,200	5	3.2%	0.00 [-1.24, 1.24]
Yang 2019 (DDDEEK) 5d E.coli	30,600	9,200	5	30,600	9,200	5	3.2%	0.00 [-1.24, 1.24]
Yang 2019 (DDDEEK) 5d E. coli tissue	1,270,000	240,000	5	1,270,000	240,000	5	3.2%	0.00 [-1.24, 1.24]
Yang 2019 (DDDEEK) 5d tissue	1,070,000	210,000	5	1,070,000	210,000	5	3.2%	0.00 [-1.24, 1.24]
Yang 2019 (WRWRWR) 5d	5	1	5	30,600	9,200	5	1.8%	-4.25 [-6.94, -1.55]
Yang 2019 (WRWRWR) 5d E.coli	5	1	5	30,600	9,200	5	1.8%	-4.25 [-6.94, -1.55]
Yang 2019 (WRWRWR) 5d E.coli tissue	5	1	5	1,270,000	240,000	5	1.1%	-6.76 [-10.76, -2.76]
Yang 2019 (WRWRWR) 5d tissue	5	1	5	1,070,000	210,000	5	1.1%	-6.51 [-10.38, -2.64]
**Subtotal (95% CI)**			**123**			**123**	**44.6%**	**-1.37 [-1.95, -0.80]**
Heterogeneity: Tau² = 0.82; Chi² = 47.14, df = 15 (P < 0.0001); I² = 68%								
Test for overall effect: Z = 4.67 (P < 0.00001)								
**1.4.3 7 Days**								
Adams 2009 (VAN) 7d	3	3	3	11,231	5,213	3	1.7%	-2.44 [-5.28, 0.41]
Chen 2016 (Cys-Mel) 7d 10^5 mice	24,200	22,700	10	326,000	516,000	10	3.5%	-0.79 [-1.71, 0.13]
Chen 2016 (Cys-Mel) 7d 10^5 mice tissue	91,800	135,000	10	899,000	1,110,000	10	3.5%	-0.98 [-1.92, -0.04]
Chen 2019 (HHC36) 7d cavity	3,600	100	3	11,600	0.06	3	0.0%	-90.51 [-177.92, -3.10]
Chen 2019 (HHC36) 7d implant	18,300	5,000	3	85,000	10,800	3	0.5%	-6.34 [-12.67, -0.01]
Chen 2020 (HHC36) 7d Ti-NT-A cavity	19,500,000	8,600,000	3	275,000,000	61,000,000	3	0.8%	-4.69 [-9.50, 0.11]
Chen 2020 (HHC36) 7d Ti-NT-A implant	95,000	180,000	3	90,000,000	30,000,000	3	1.2%	-3.39 [-7.03, 0.25]
Chen 2021 (HHC36) 7d	667	328	3	138,000	18,000	3	0.3%	-8.63 [-17.12, -0.14]
Chen 2021 (QK, HHC36) 7d Ti-125FP	780	510	3	138,000	18,000	3	0.3%	-8.62 [-17.10, -0.14]
Chen 2021 (QK) 7d	179,000	68,000	3	138,000	18,000	3	2.6%	0.66 [-1.06, 2.38]
Fang 2021 (HHC36) 7d	1,270	415	3	76,800	13,600	3	0.5%	-6.28 [-12.55, -0.01]
Fang 2021 (RGD) 7d	80,900	7,970	3	76,800	13,600	3	2.7%	0.29 [-1.33, 1.92]
**Subtotal (95% CI)**			**50**			**50**	**17.6%**	**-1.60 [-2.78, -0.42]**
Heterogeneity: Tau² = 1.65; Chi² = 26.69, df = 11 (P = 0.005); I² = 59%								
Test for overall effect: Z = 2.66 (P = 0.008)								
**1.4.4 14 Days**								
Adams 2009 (VAN) 14d	1.57	1.14	3	2,186	3,003	3	2.6%	-0.82 [-2.61, 0.96]
**Subtotal (95% CI)**			**3**			**3**	**2.6%**	**-0.82 [-2.61, 0.96]**
Heterogeneity: Not applicable								
Test for overall effect: Z = 0.90 (P = 0.37)								
**1.4.5 21 Days**								
Adams 2009 (VAN) 21d	12,989	13,144	3	177,891	223,658	3	2.6%	-0.83 [-2.62, 0.96]
Nie 2017 (Bacitracin) 21d bone tissue	6,010	3,230	5	289,000	143,000	5	2.5%	-2.53 [-4.41, -0.64]
Nie 2017 (Bacitracin) 21d implant	3,130	1,670	5	467,000	234,000	5	2.5%	-2.53 [-4.42, -0.64]
Zhang 2019 (VAN) 21d	68	71.2	7	1,490	533	7	2.5%	-3.50 [-5.36, -1.65]
**Subtotal (95% CI)**			**20**			**20**	**10.0%**	**-2.33 [-3.44, -1.22]**
Heterogeneity: Tau² = 0.39; Chi² = 4.30, df = 3 (P = 0.23); I² = 30%								
Test for overall effect: Z = 4.11 (P < 0.0001)								
**1.4.6 28 Days**								
de Breij 2016 (OP-145) 28 days tissue	2,290	4,580	5	672,000	1,340,000	7	3.2%	-0.60 [-1.78, 0.59]
de Breij 2016 (OP-145) 28d bone	3,970,000	8,850,000	6	7,360,000	10,200,000	7	3.3%	-0.33 [-1.43, 0.77]
de Breij 2016 (OP-145) 28d nail	301,000	597,000	6	58,100	130,000	7	3.3%	0.55 [-0.57, 1.67]
**Subtotal (95% CI)**			**17**			**21**	**9.8%**	**-0.11 [-0.78, 0.56]**
Heterogeneity: Tau² = 0.02; Chi² = 2.12, df = 2 (P = 0.35); I² = 6%								
Test for overall effect: Z = 0.33 (P = 0.74)								
**1.4.7 42 Days**								
Stavrakis 2019 (VAN) 42d bone	2	2	6	370,000	110,000	6	2.0%	-4.39 [-6.81, -1.97]
Stavrakis 2019 (VAN) 42d implant	1	1	6	280	150	6	2.7%	-2.43 [-4.07, -0.79]
Zhang 2018 (VAN) 42d implant	40,400	8,900	10	842,000	68,000	10	0.6%	-15.83 [-21.38, -10.29]
Zhang 2018 (VAN) 42d tibia	3,040	370	10	32,400	3,800	10	1.2%	-10.42 [-14.12, -6.71]
**Subtotal (95% CI)**			**32**			**32**	**6.5%**	**-7.68 [-12.38, -2.98]**
Heterogeneity: Tau² = 19.88; Chi² = 31.97, df = 3 (P < 0.00001); I² = 91%								
Test for overall effect: Z = 3.20 (P = 0.001)								
**1.4.8 12 Weeks**								
Sinclair 2013 (CSA-13) 12w	23.3	52.2	5	114,000	144,000	3	2.7%	-1.19 [-2.84, 0.46]
**Subtotal (95% CI)**			**5**			**3**	**2.7%**	**-1.19 [-2.84, 0.46]**
Heterogeneity: Not applicable								
Test for overall effect: Z = 1.42 (P = 0.16)								
**Total (95% CI)**			**266**			**268**	**100.0%**	**-1.74 [-2.22, -1.26]**
Heterogeneity: Tau² = 1.53; Chi² = 159.76, df = 42 (P < 0.00001); I² = 74%								
Test for overall effect: Z = 7.05 (P < 0.00001)								
Test for subgroup differences: Chi² = 23.28, df = 7 (P = 0.002), I² = 69.9%								

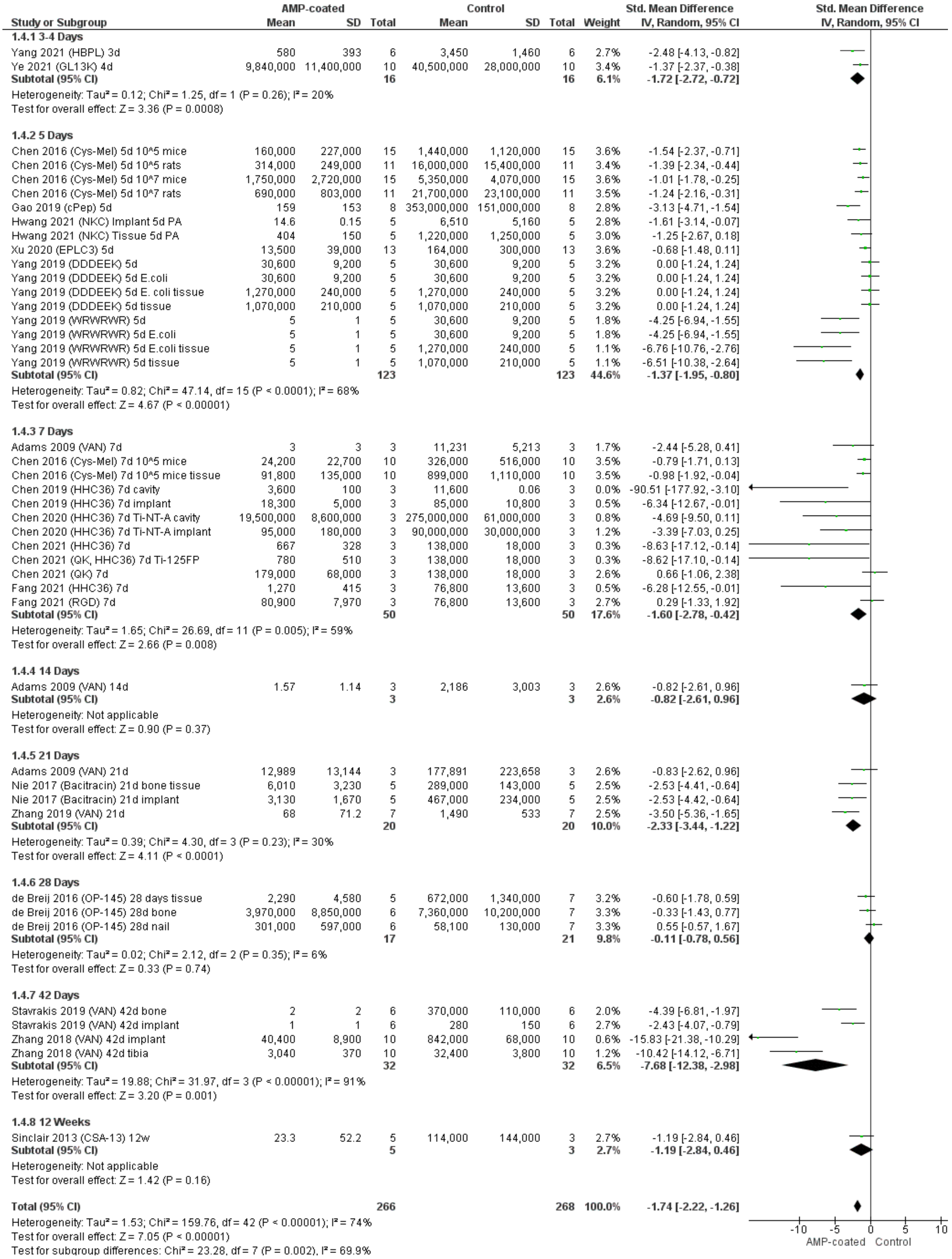

Figure S4: Forest plot of subgroup meta-anlysis for assessment of AMP-coated implants on bacterial infection prevention as CFU counts expression, considering sample type. Mean, standard deviation and sample size taken from 18 studies comparing effect size using Hedge's g as the meta-analysis. Effect size shown comparing control and AMP-coated groups with 95% Confidence Intervals and overall effect size as a diamond. Heterogeneity between studies, Tau^2, Chi^2 and I^2 were also calculated ($P<0.00001$). Subgroup analysis further compares overall effects sizes for different culture samples taken. IV, inverse variance. All bacteria used were *S. aureus* unless indicated otherwise (*E. coli* or *P. aeruginosa* (PA))

.

	AMP-coated			Control				Std. Mean Difference
Study or Subgroup	Mean	SD	Total	Mean	SD	Total	Weight	IV, Random, 95% CI
**1.5.1 Implant**								
Adams 2009 (VAN) 14d	1.57	1.14	3	2,186	3,003	3	2.6%	-0.82 [-2.61, 0.96]
Adams 2009 (VAN) 21d	12,989	13,144	3	177,891	223,658	3	2.6%	-0.83 [-2.62, 0.96]
Adams 2009 (VAN) 7d	3	3	3	11,231	5,213	3	1.7%	-2.44 [-5.28, 0.41]
Chen 2016 (Cys-Mel) 5d 10^5 mice	160,000	227,000	15	1,440,000	1,120,000	15	3.6%	-1.54 [-2.37, -0.71]
Chen 2016 (Cys-Mel) 5d 10^5 rats	314,000	249,000	11	16,000,000	15,400,000	11	3.4%	-1.39 [-2.34, -0.44]
Chen 2016 (Cys-Mel) 5d 10^7 mice	1,750,000	2,720,000	15	5,350,000	4,070,000	15	3.6%	-1.01 [-1.78, -0.25]
Chen 2016 (Cys-Mel) 5d 10^7 rats	690,000	803,000	11	21,700,000	23,100,000	11	3.5%	-1.24 [-2.16, -0.31]
Chen 2016 (Cys-Mel) 7d 10^5 mice	24,200	22,700	10	326,000	516,000	10	3.5%	-0.79 [-1.71, 0.13]
Chen 2019 (HHC36) 7d implant	18,300	5,000	3	85,000	10,800	3	0.5%	-6.34 [-12.67, -0.01]
Chen 2020 (HHC36) 7d Ti-NT-A implant	95,000	180,000	3	90,000,000	30,000,000	3	1.2%	-3.39 [-7.03, 0.25]
Chen 2021 (HHC36) 7d	667	328	3	138,000	18,000	3	0.3%	-8.63 [-17.12, -0.14]
Chen 2021 (QK, HHC36) 7d Ti-125FP	780	510	3	138,000	18,000	3	0.3%	-8.62 [-17.10, -0.14]
Chen 2021 (QK) 7d	179,000	68,000	3	138,000	18,000	3	2.6%	0.66 [-1.06, 2.38]
de Breij 2016 (OP-145) 28d nail	301,000	597,000	6	58,100	130,000	7	3.3%	0.55 [-0.57, 1.67]
Fang 2021 (HHC36) 7d	1,270	415	3	76,800	13,600	3	0.5%	-6.28 [-12.55, -0.01]
Fang 2021 (RGD) 7d	80,900	7,970	3	76,800	13,600	3	2.7%	0.29 [-1.33, 1.92]
Gao 2019 (cPep) 5d	159	153	8	353,000,000	151,000,000	8	2.8%	-3.13 [-4.71, -1.54]
Hwang 2021 (NKC) Implant 5d PA	14.6	0.15	5	6,510	5,160	5	2.8%	-1.61 [-3.14, -0.07]
Nie 2017 (Bacitracin) 21d implant	3,130	1,670	5	467,000	234,000	5	2.5%	-2.53 [-4.42, -0.64]
Sinclair 2013 (CSA-13) 12w	23.3	52.2	5	114,000	144,000	3	2.7%	-1.19 [-2.84, 0.46]
Stavrakis 2019 (VAN) 42d implant	1	1	6	280	150	6	2.7%	-2.43 [-4.07, -0.79]
Xu 2020 (EPLC3) 5d	13,500	39,000	13	164,000	300,000	13	3.6%	-0.68 [-1.48, 0.11]
Yang 2019 (DDDEEK) 5d	30,600	9,200	5	30,600	9,200	5	3.2%	0.00 [-1.24, 1.24]
Yang 2019 (DDDEEK) 5d E.coli	30,600	9,200	5	30,600	9,200	5	3.2%	0.00 [-1.24, 1.24]
Yang 2019 (WRWRWR) 5d	5	1	5	30,600	9,200	5	1.8%	-4.25 [-6.94, -1.55]
Yang 2019 (WRWRWR) 5d E.coli	5	1	5	30,600	9,200	5	1.8%	-4.25 [-6.94, -1.55]
Yang 2021 (HBPL) 3d	580	393	6	3,450	1,460	6	2.7%	-2.48 [-4.13, -0.82]
Ye 2021 (GL13K) 4d	9,840,000	11,400,000	10	40,500,000	28,000,000	10	3.4%	-1.37 [-2.37, -0.38]
Zhang 2018 (VAN) 42d implant	40,400	8,900	10	842,000	68,000	10	0.6%	-15.83 [-21.38, -10.29]
Zhang 2019 (VAN) 21d	68	71.2	7	1,490	533	7	2.5%	-3.50 [-5.36, -1.65]
**Subtotal (95% CI)**			**193**			**192**	**72.1%**	**-1.57 [-2.09, -1.05]**
Heterogeneity: Tau² = 1.18; Chi² = 96.00, df = 29 (P < 0.00001); I² = 70%								
Test for overall effect: Z = 5.93 (P < 0.00001)								
**1.5.2 Surrounding Tissue**								
Chen 2016 (Cys-Mel) 7d 10^5 mice tissue	91,800	135,000	10	899,000	1,110,000	10	3.5%	-0.98 [-1.92, -0.04]
Chen 2019 (HHC36) 7d cavity	3,600	100	3	11,600	0.06	3	0.0%	-90.51 [-177.92, -3.10]
Chen 2020 (HHC36) 7d Ti-NT-A cavity	19,500,000	8,600,000	3	275,000,000	61,000,000	3	0.8%	-4.69 [-9.50, 0.11]
de Breij 2016 (OP-145) 28 days tissue	2,290	4,580	5	672,000	1,340,000	7	3.2%	-0.60 [-1.78, 0.59]
de Breij 2016 (OP-145) 28d bone	3,970,000	8,850,000	6	7,360,000	10,200,000	7	3.3%	-0.33 [-1.43, 0.77]
Hwang 2021 (NKC) Tissue 5d PA	404	150	5	1,220,000	1,250,000	5	3.0%	-1.25 [-2.67, 0.18]
Nie 2017 (Bacitracin) 21d bone tissue	6,010	3,230	5	289,000	143,000	5	2.5%	-2.53 [-4.41, -0.64]
Stavrakis 2019 (VAN) 42d bone	2	2	6	370,000	110,000	6	2.0%	-4.39 [-6.81, -1.97]
Yang 2019 (DDDEEK) 5d E. coli tissue	1,270,000	240,000	5	1,270,000	240,000	5	3.2%	0.00 [-1.24, 1.24]
Yang 2019 (DDDEEK) 5d tissue	1,070,000	210,000	5	1,070,000	210,000	5	3.2%	0.00 [-1.24, 1.24]
Yang 2019 (WRWRWR) 5d E.coli tissue	5	1	5	1,270,000	240,000	5	1.1%	-6.76 [-10.76, -2.76]
Yang 2019 (WRWRWR) 5d tissue	5	1	5	1,070,000	210,000	5	1.1%	-6.51 [-10.38, -2.64]
Zhang 2018 (VAN) 42d tibia	3,040	370	10	32,400	3,800	10	1.2%	-10.42 [-14.12, -6.71]
**Subtotal (95% CI)**			**73**			**76**	**27.9%**	**-2.35 [-3.52, -1.18]**
Heterogeneity: Tau² = 3.02; Chi² = 63.63, df = 12 (P < 0.00001); I² = 81%								
Test for overall effect: Z = 3.93 (P < 0.0001)								
**Total (95% CI)**			**266**			**268**	**100.0%**	**-1.74 [-2.22, -1.26]**
Heterogeneity: Tau² = 1.53; Chi² = 159.76, df = 42 (P < 0.00001); I² = 74%								
Test for overall effect: Z = 7.05 (P < 0.00001)								
Test for subgroup differences: Chi² = 1.41, df = 1 (P = 0.24), I² = 29.0%								

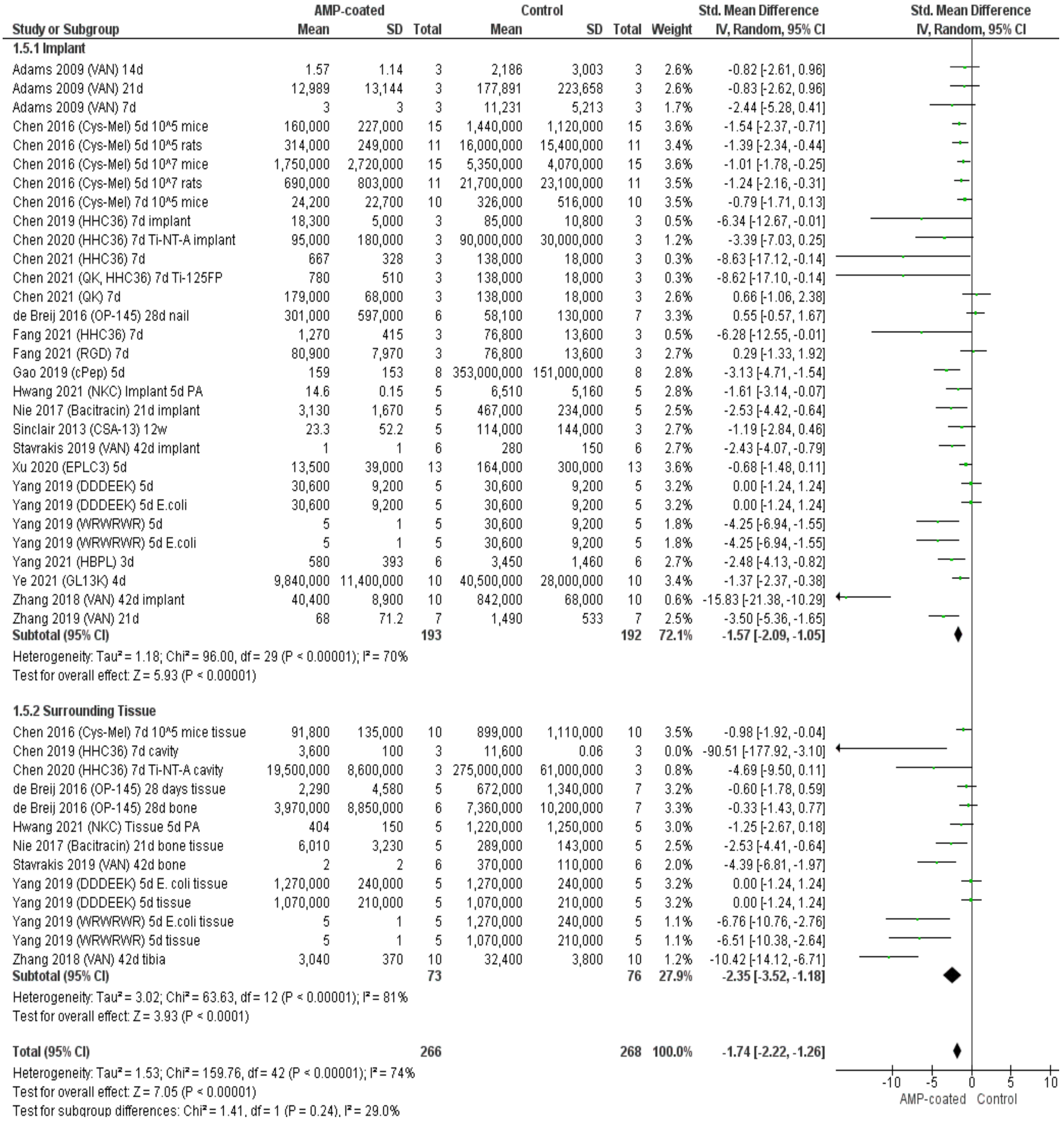

Supplement: Supplementary file 1 — ab1c01307_si_001.pdf [file ab1c01307_si_001.pdf]
